# Supplementary material for: Regulating interfacial reaction through electrolyte chemistry enables gradient interphase for low-temperature zinc metal batteries
Source: Nat Commun. 2023 Sep 6;14:5443. doi: 10.1038/s41467-023-41276-9 (PMC10482877; doi:10.1038/s41467-023-41276-9)
Supplement: Supplementary file 1 — Supplementary Information [file 41467_2023_41276_MOESM1_ESM.pdf]

Supporting Information

Regulating Interfacial Reaction through Electrolyte Chemistry Enables Gradient Interphase for Low-Temperature Zinc Metal Batteries

Wei Wang[1]*, Shan Chen*[1]*, Xuelong Liao*[1]*, Rong Huang*[1]*, Fengmei Wang*[2]*, Jialei Chen*[1]*, Yaxin Wang*[1]*, Fei Wang*[2]**, Huan Wang*[1]**

[1]Key Laboratory of Advanced Energy Materials Chemistry (Ministry of Education), Renewable Energy Conversion and Storage Center (RECAST), College of Chemistry, Nankai University, Tianjin 300071, China

[2]Department of Materials Science, Fudan University, Shanghai 200433, China

*E-mail: huan.wang0520@nankai.edu.cn; feiw@fudan.edu.cn

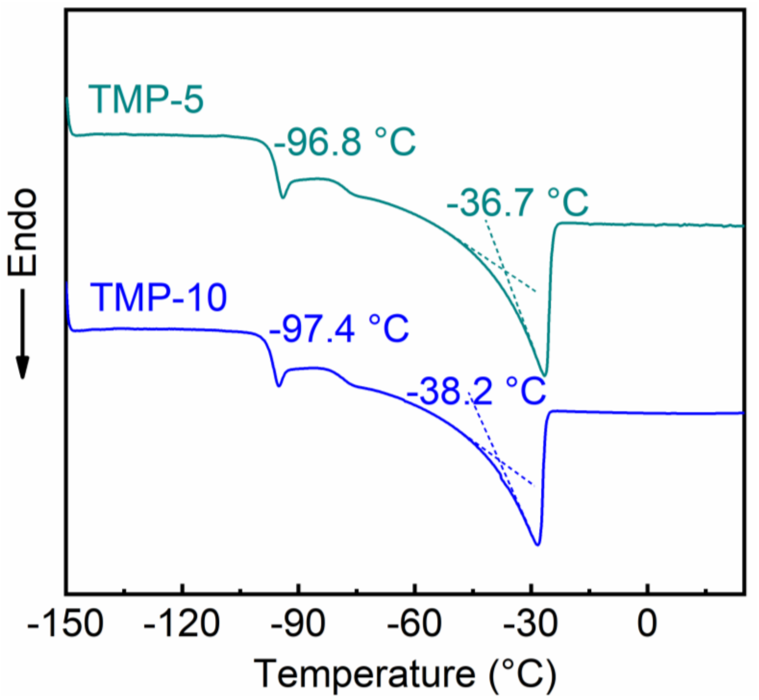

Supplementary Figure 1. Differential scanning calorimetry (DSC) curves of TMP–5 and TMP–10 electrolytes.

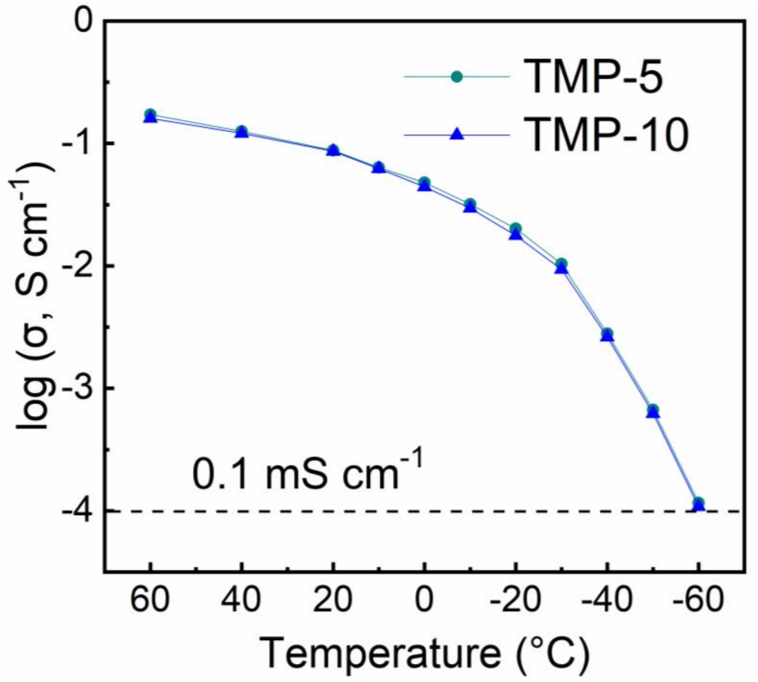

Supplementary Figure 2. Ionic conductivity of TMP–5 and TMP–10 electrolytes at different temperatures.

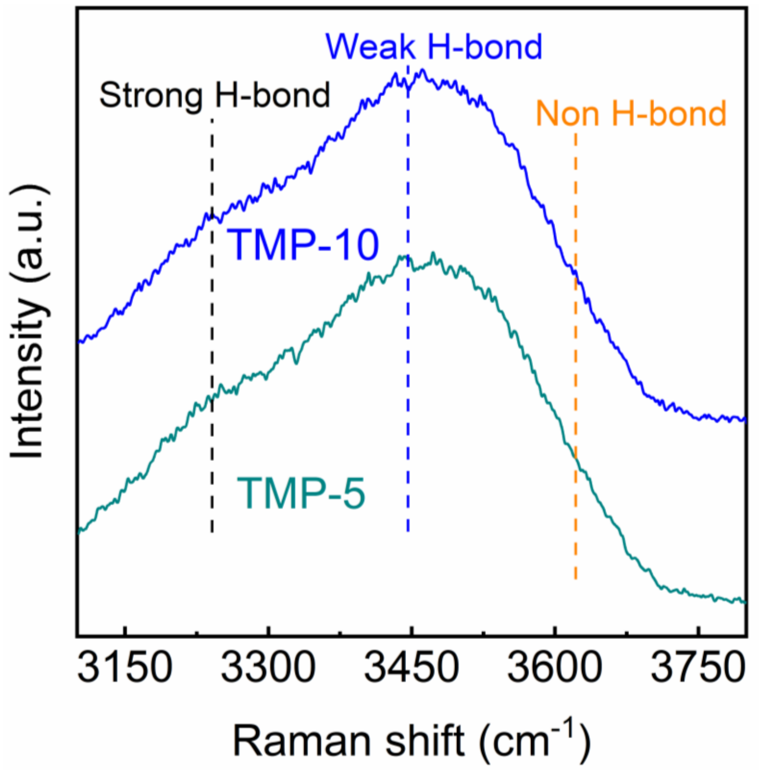

Supplementary Figure 3. Raman spectra of O–H stretching vibration in TMP–5 and TMP–10 electrolytes.

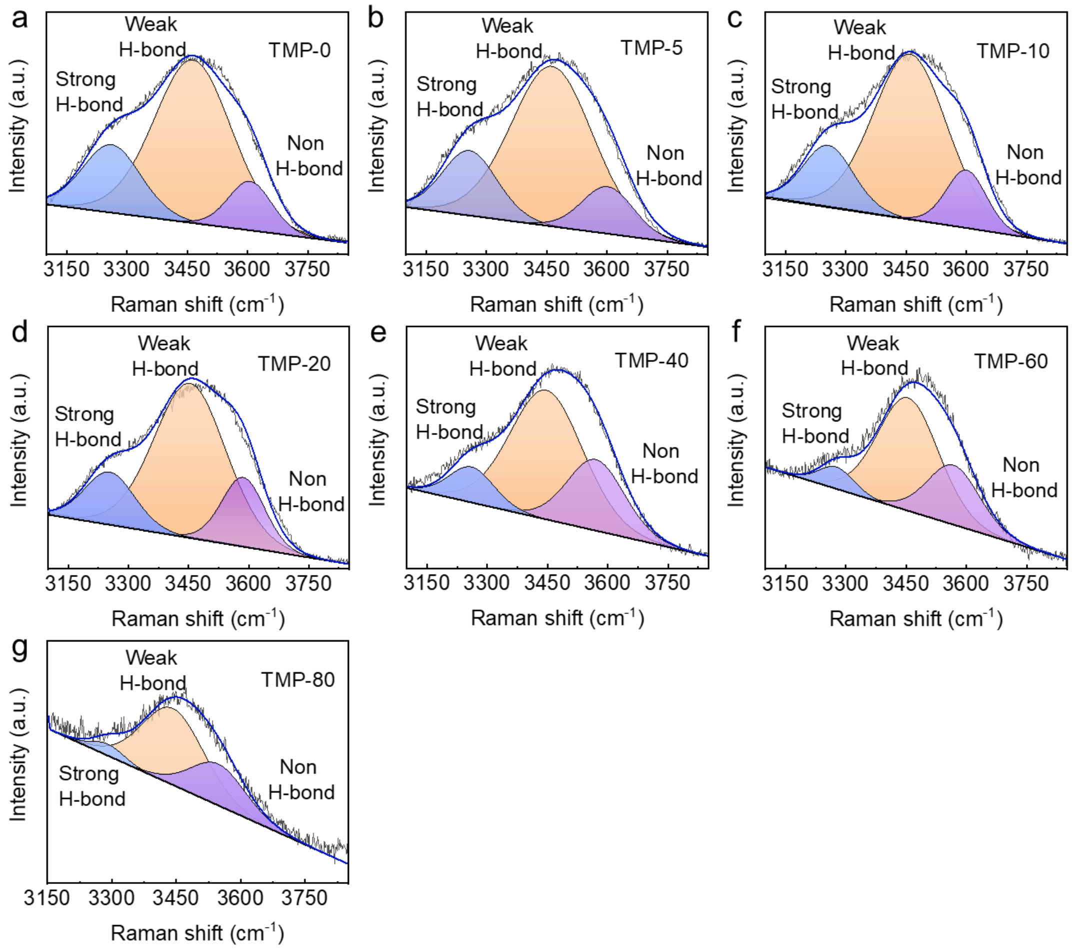

Supplementary Figure 4. The fitted H-bonds in various hybrids of electrolytes. (a) TMP–0, (b) TMP–5, (c) TMP–10, (d) TMP–20, (e) TMP–40, (f) TMP–60 and (g) TMP–80.

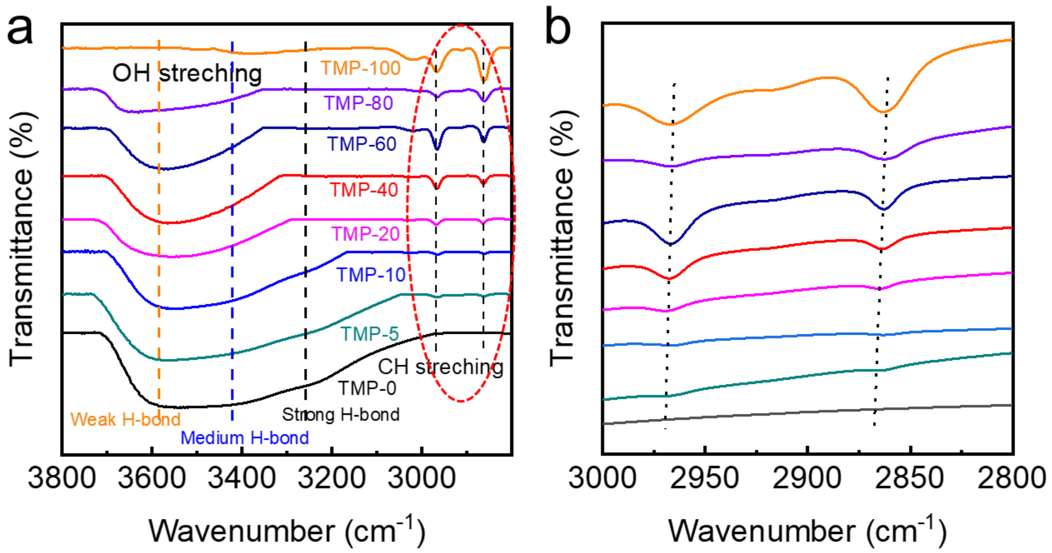

Supplementary Figure 5. FTIR analysis of different hybrid electrolytes. (a) Overall spectra and (b) the enlarged view of the selected region indicated by a red oval box.

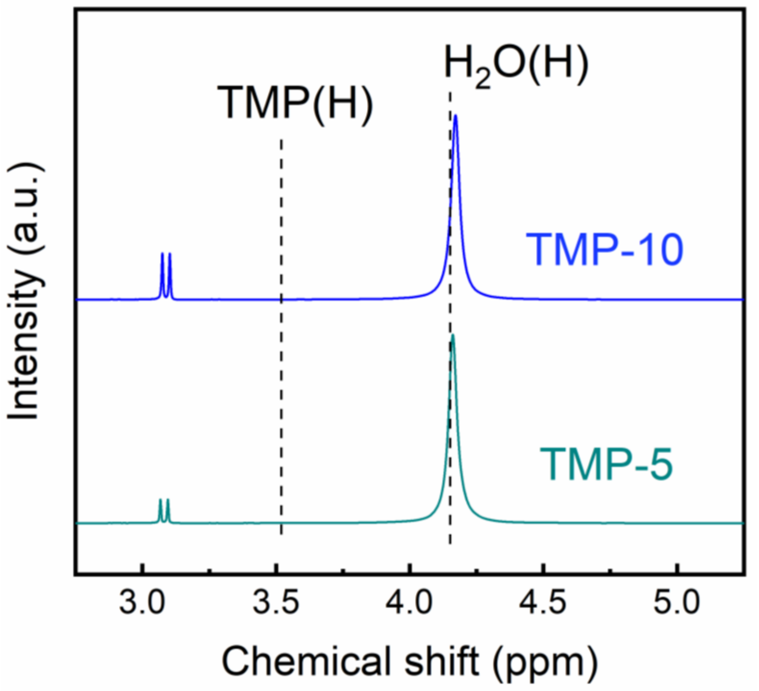

Supplementary Figure 6. ^{1}H NMR spectra of TMP–5 and TMP–10 electrolytes.

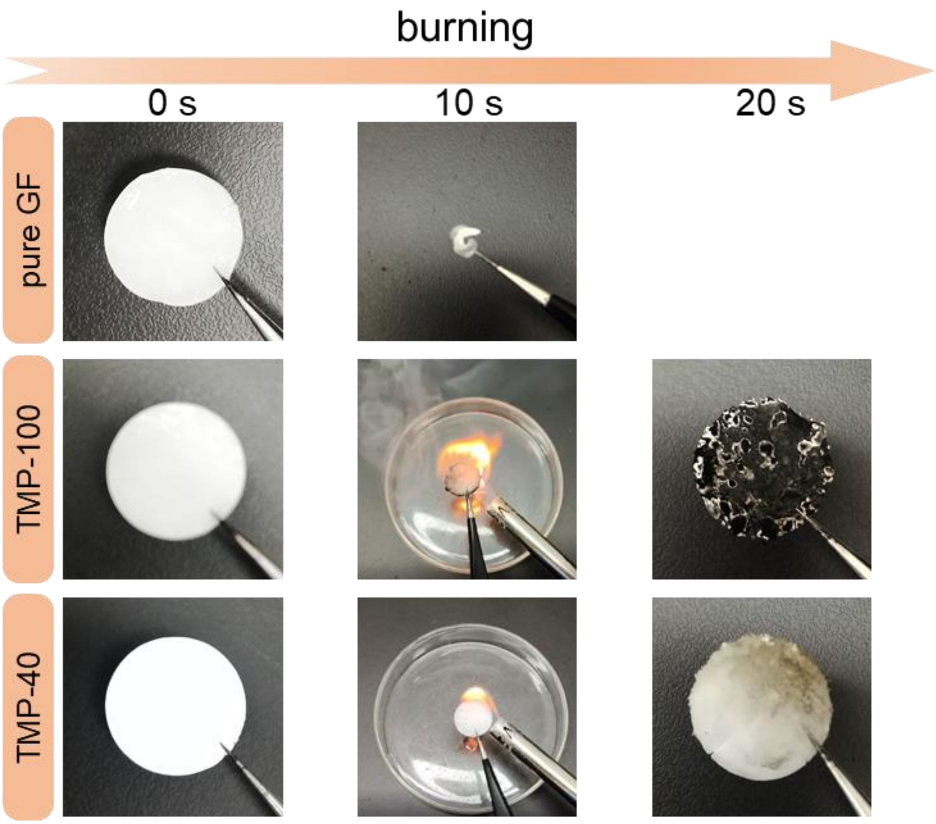

Supplementary Figure 7. Photographs of the combustion tests of pure glass fiber (pure GF) (top), glass fiber rinsed with TMP–100 (middle) and TMP–40 (bottom) electrolytes.

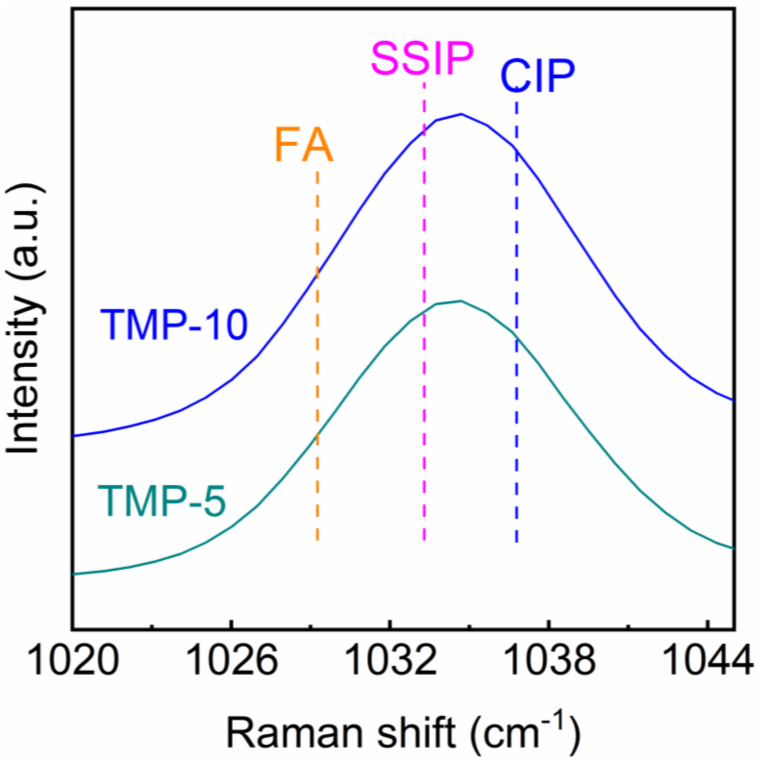

Supplementary Figure 8. Raman spectra to reveal the SO_3 stretching mode in TMP–5 and TMP–10 electrolytes.

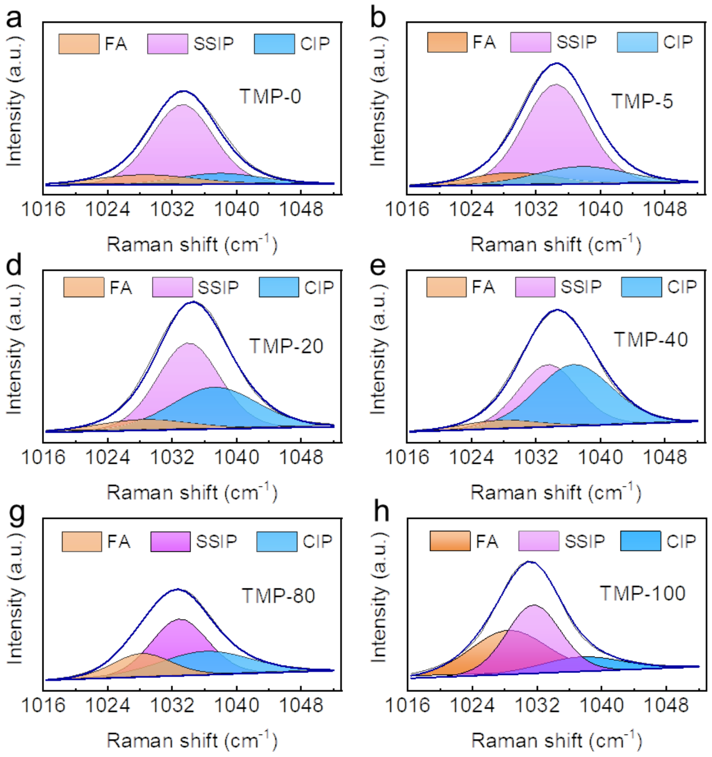

Supplementary Figure 9. The fitted SO_3 stretching vibration in various kinds of hybrid electrolytes. FA, SSIP and CIP stand for free anion (OTf^-), solvent-separated ion pairs (Zn^{2+}–$(H_2O)_x(TMP)_y$–OTf^-) and contact ion pairs (Zn^{2+}–OTf^-), respectively. (a) TMP–0, (b) TMP–5, (c) TMP–10, (d) TMP–20, (e) TMP–40, (f) TMP–60, (g) TMP–80 and (h) TMP–100.

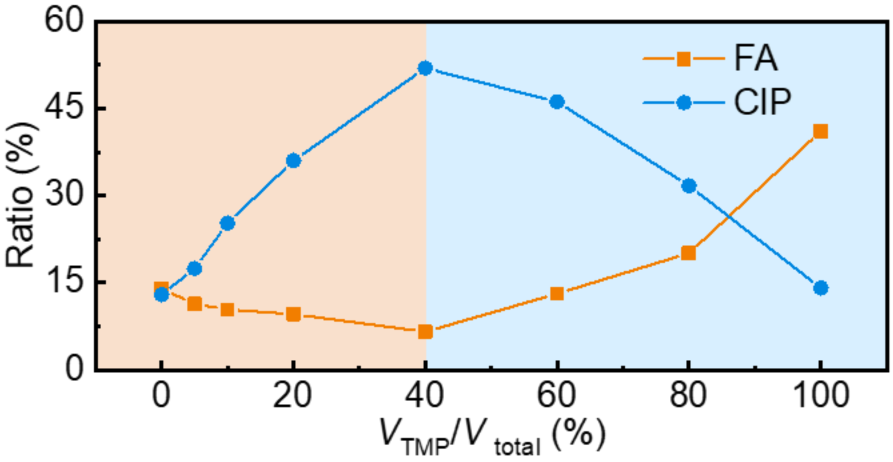

Supplementary Figure 10. The FA and CIP ratios with TMP content.

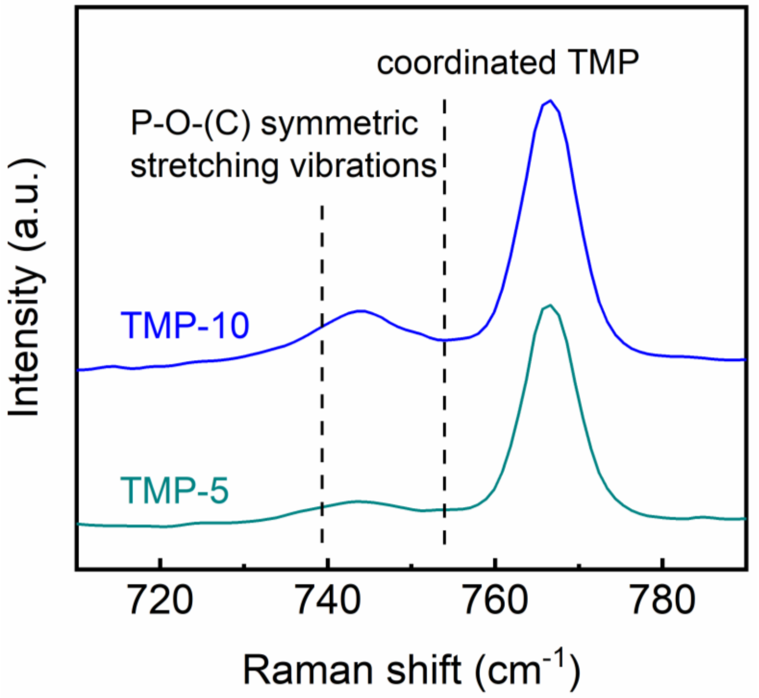

Supplementary Figure 11. Raman spectra to reveal the P–O–(C) symmetric stretching vibration mode in TMP–5 and TMP–10 electrolytes.

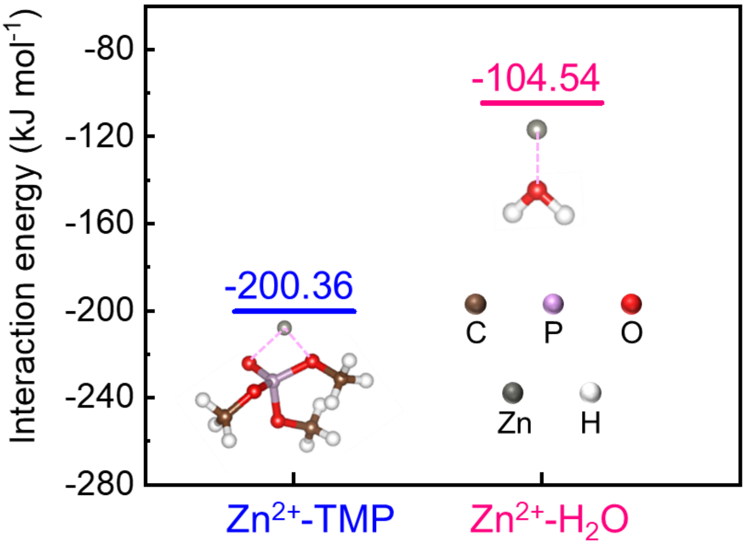

Supplementary Figure 12. The binding energy of Zn^{2+}–TMP and Zn^{2+}–H_2O obtained from DFT calculations.

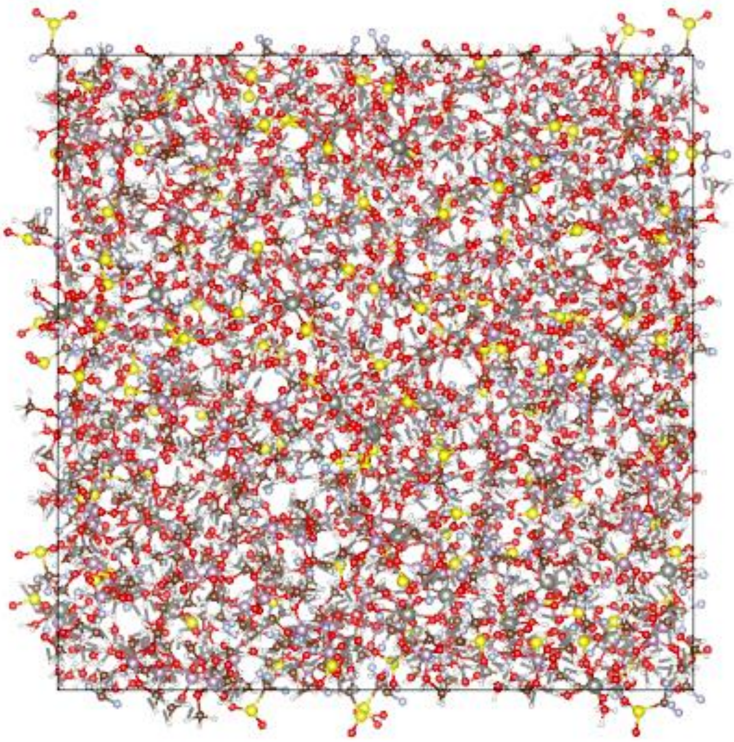

Supplementary Figure 13. Snapshot of the MD simulation cell for the TMP–40 electrolyte.

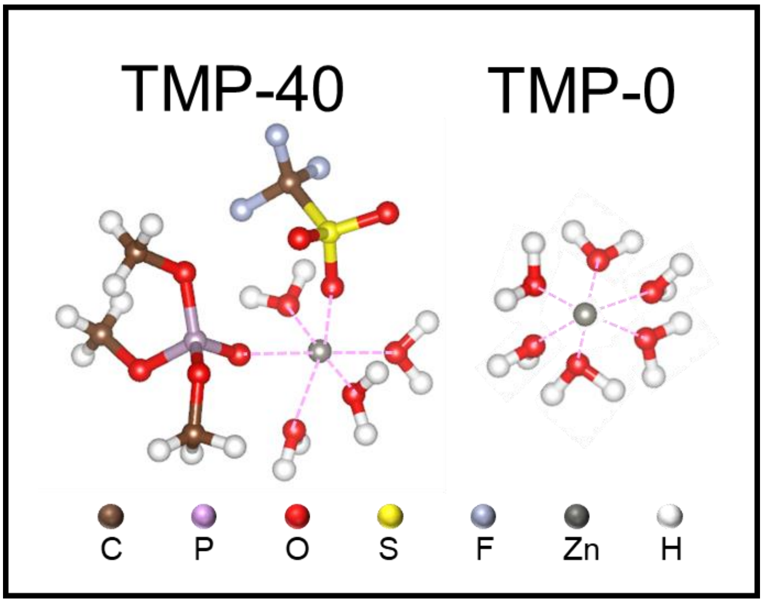

Supplementary Figure 14. Solvation structure model of TMP–0 and TMP–40 electrolytes.

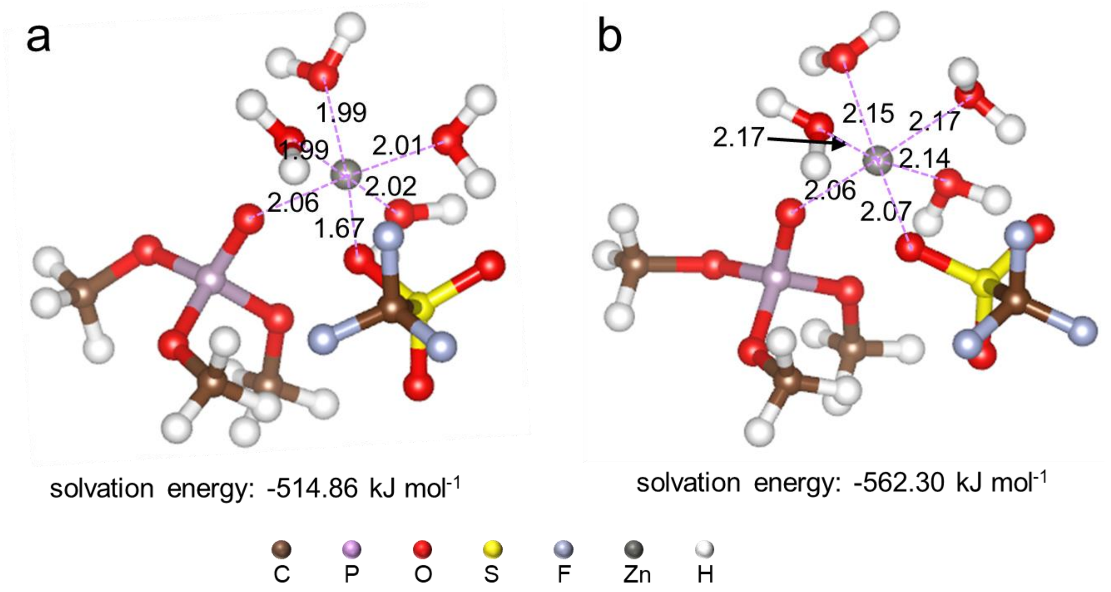

Supplementary Figure 15. Optimized geometric configurations (distance in Å) and solvation energies of the representative Zn(OTf)(TMP)$(H_2O)_4$ cluster with different calculation methods. (a) PCFF-INTERFACE force field and (b) B3LYP/6-31++G (d, p).

The solvation energies predicated by the two simulation methods are only deviated by 8.4% (−514. 86 kJ mol^{-1} vs. −562.30 kJ mol^{-1}). In the prediction of solvation sheath geometries, except for a certain deviation (0.4 Å) in the distance between Zn^{2+} and OTF^{-}, other structural parameters are consistent. Therefore, considering the approximation of the force field, the molecular dynamics research results using PCFF-INTERFACE force field in our system research are acceptable.

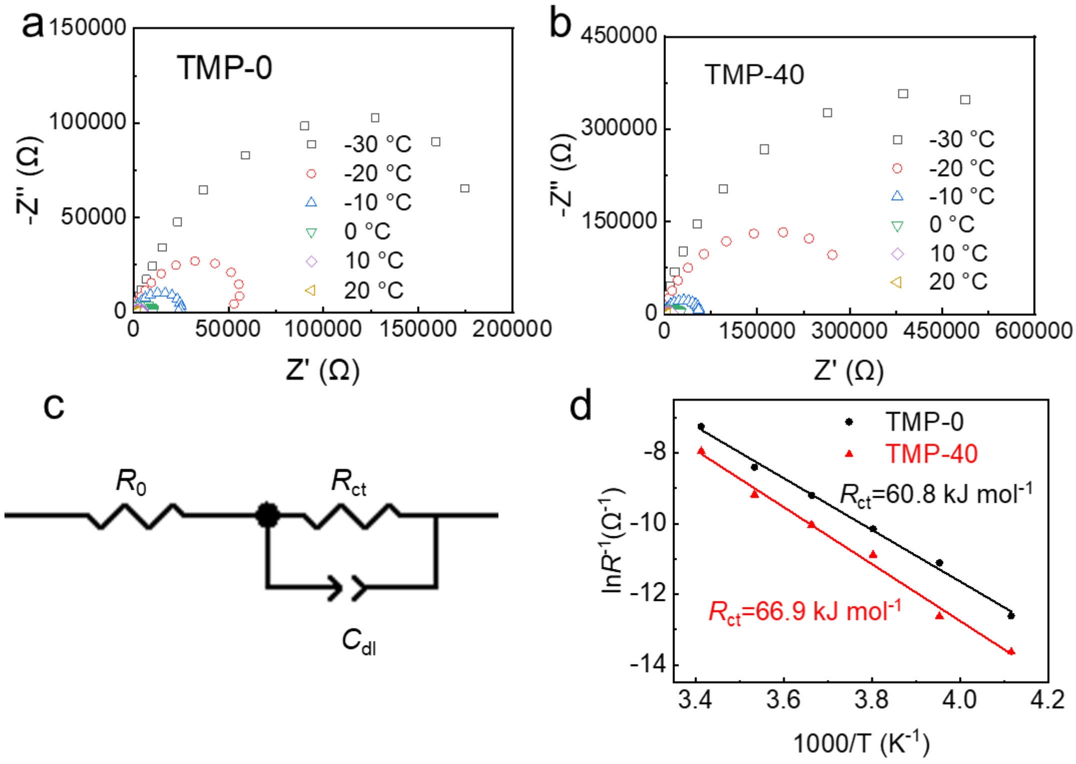

The fitting results corresponding to R_{ct} are as follows:

Temperature (K)		243	253	263	273	283	293
TMP–0	Rct (Ω)	299900	66934	25552	9941	4477	1408
TMP–40	Rct (Ω)	827930	302790	53342	22825	9720	2837

Supplementary Figure 16. Comparison of desolvation energy of TMP–0 and TMP–40 electrolytes before cycling. Temperature-dependent electrochemical impedance spectra of Zn||Zn symmetric cells with (a) TMP–0 or (b) TMP–40 electrolyte. (c) The equivalent circuit model. R_0 is bulk resistance of the cell, which reflects electric conductivity of the electrolyte, separator and electrodes; R_{ct} and C_{dl} are faradic charge-transfer resistance and its relative double-layer capacitance, respectively. (d) Arrhenius fitting of R_{ct} derived from the Nyquist plots of the Zn||Zn symmetric cells with TMP–0 and TMP–40 electrolytes.

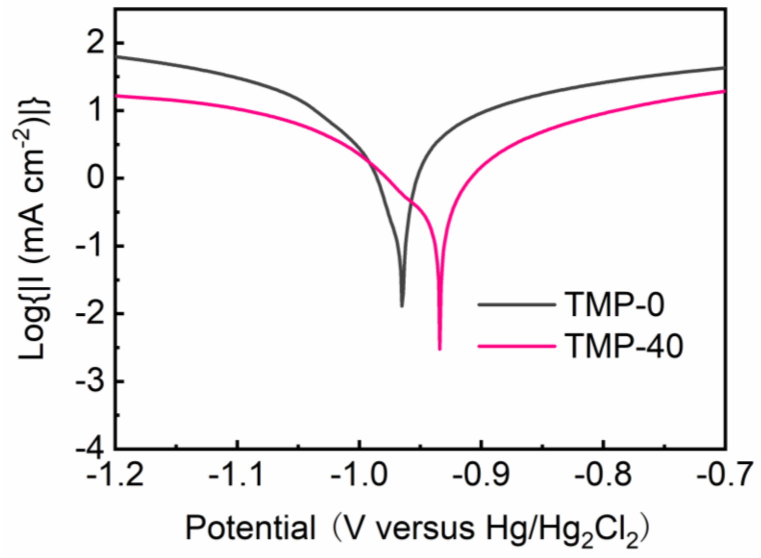

Supplementary Figure 17. Polarization curves of TMP–0 and TMP–40 electrolytes.

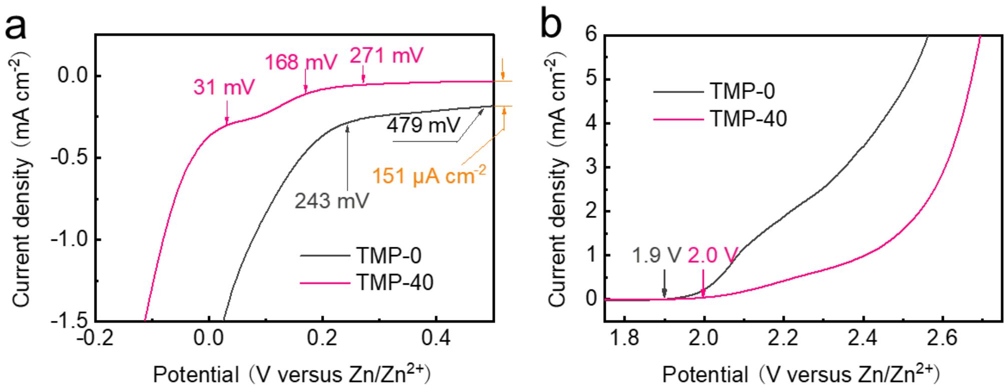

Supplementary Figure 18. Comparison of electrochemical stability windows of TMP–0 and TMP–40 electrolytes. LSV curves of TMP–0 and TMP–40 electrolytes to reveal the electrochemical stability in the voltage of (a) −0.2~0.5 V and (b) 1.75~2.75 V.

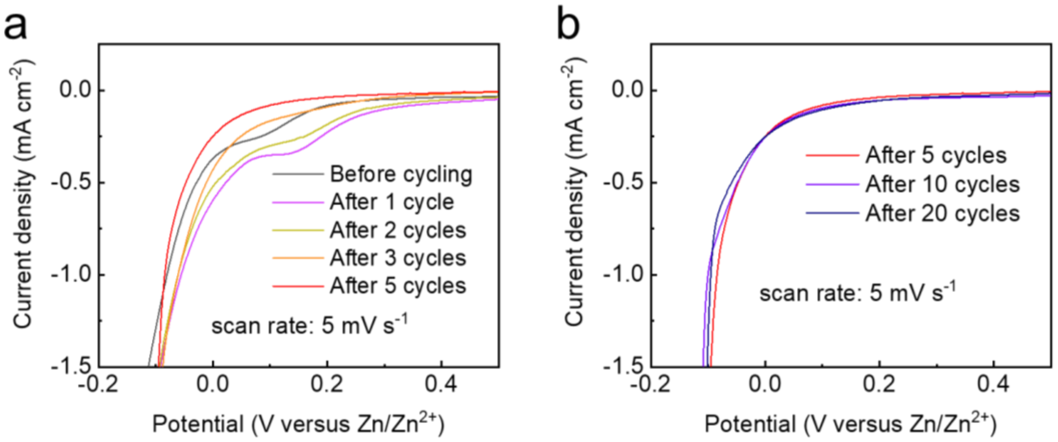

Supplementary Figure 19. LSV curves of Ti||Zn asymmetric cells after different cycles with TMP–40 electrolyte at a scan rate of 5 mV s^{-1}. (a) Within 5 cycles. (b) After 5, 10 and 20 cycles.

As shown in Supplementary Fig. 19a, the peak current density after one cycle of activation reaches the maximum value of 0.334 mA cm^{-2} and gradual decreases as the cycling proceeds. At the 5 cycles, the peak current density almost disappears and remains almost unchanged even after 20 cycles (Supplementary Fig. 19b), representing the SEI formation reaches the steady state. Also, an obviously negative shift is observed for the onset potential of hydrogen evolution reaction (HER) after 5 cycles compared to that before cycling, indicating the as-formed SEI can effectively suppress HER. These results indicate the as-formed SEI remains stable after 5 cycles.

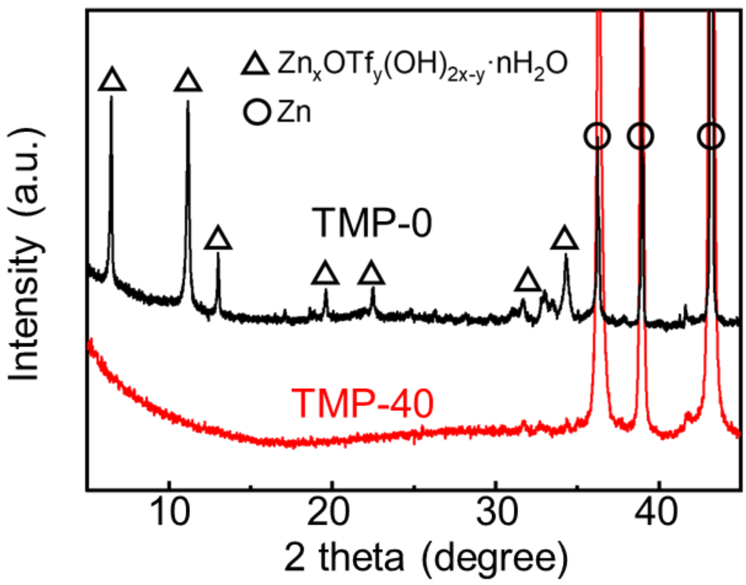

Supplementary Figure 20. XRD patterns of Zn anodes in TMP–0 and TMP–40 electrolytes after 40 cycles, where the characteristic peaks of $Zn_xOTf_y(OH)_{2x-y}\cdot nH_2O$ (ZOTH) are observed.

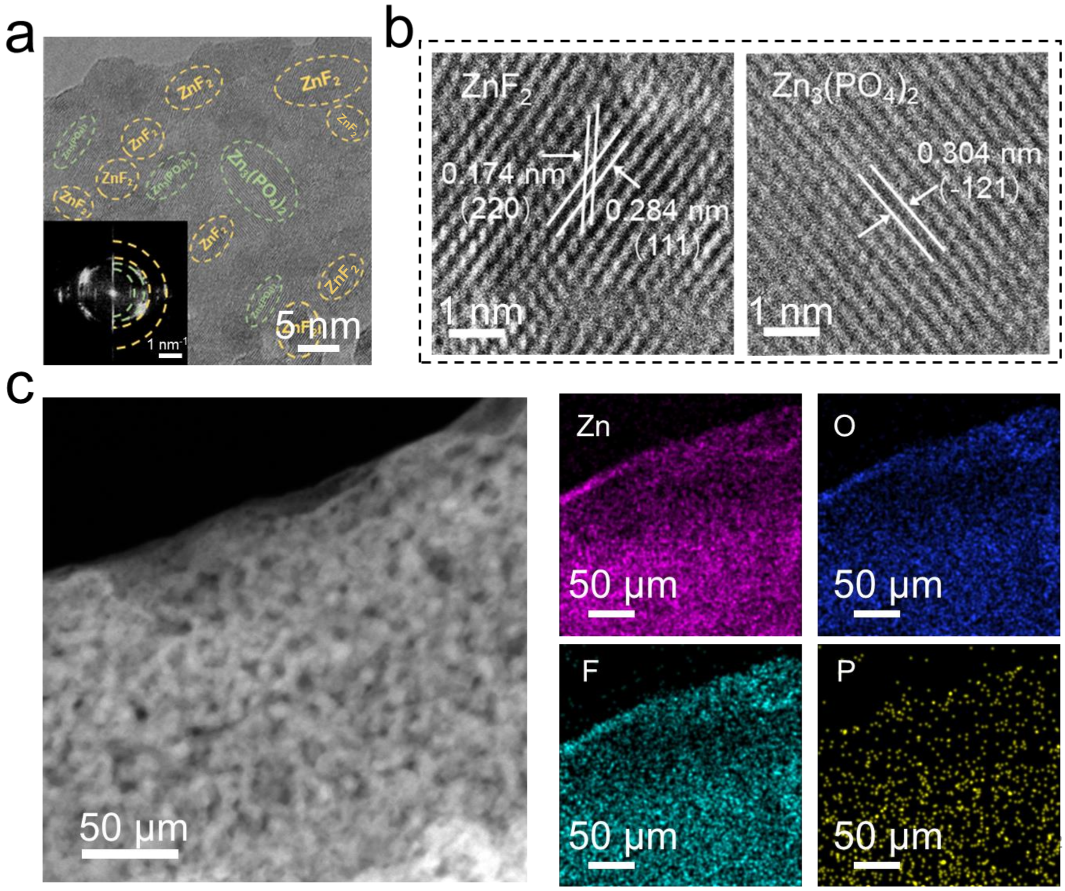

Supplementary Figure 21. TEM characterizations of Zn surface after cycling with TMP–40 electrolyte. (a) HRTEM image of Zn surface after cycling with TMP–40 electrolyte. Inset: the corresponding SAED pattern. (b) HRTEM images to reveal the lattice spacings of corresponding ZnF_2 and $Zn_3(PO_4)_2$. (c) Element distributions of SEI formed on the Zn surface in TMP–40 electrolyte.

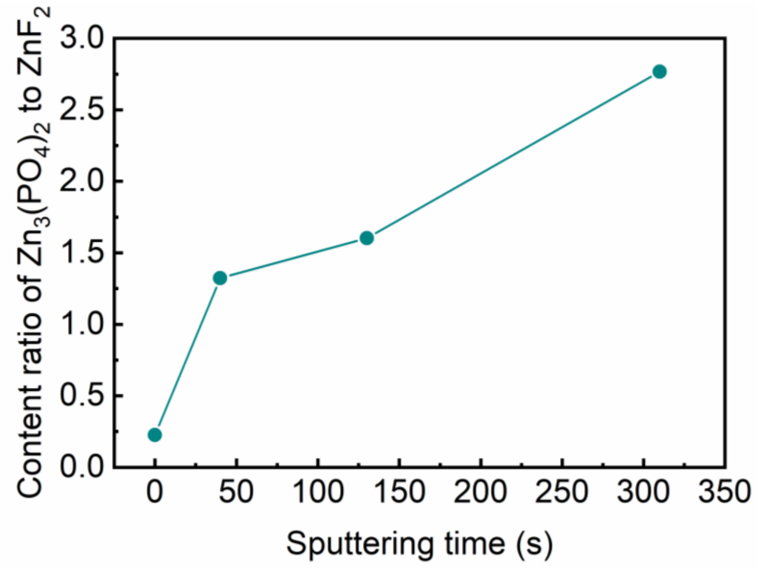

Supplementary Figure 22. The content ratio of $Zn_3(PO_4)_2$ to ZnF_2 with the increase of sputtering time.

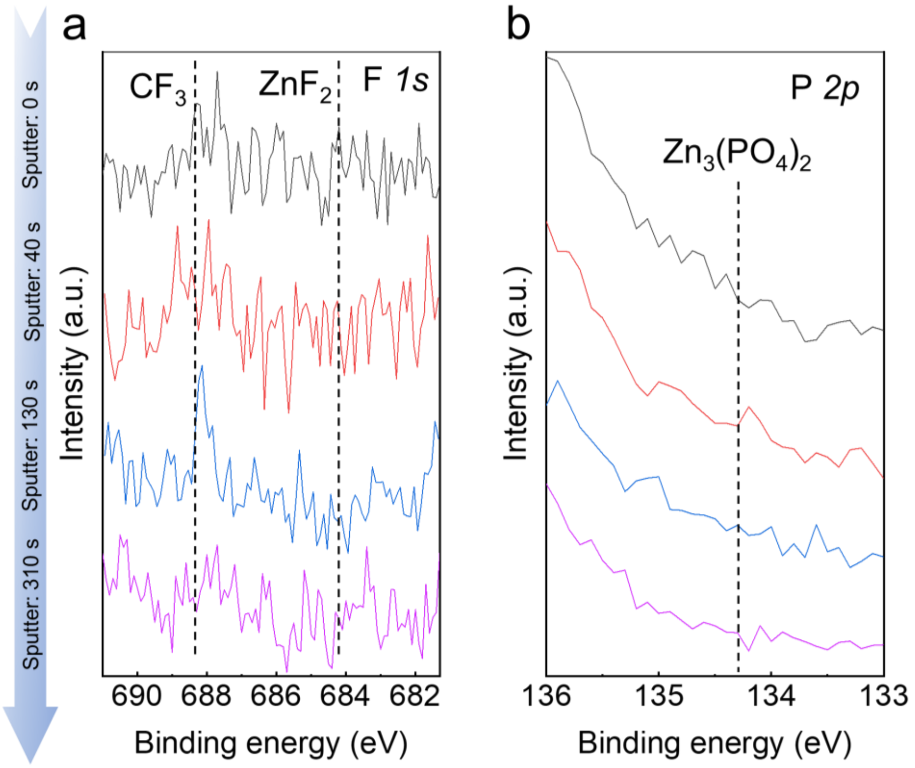

Supplementary Figure 23. XPS spectra on the Zn surface after cycling with TMP–0 electrolyte. (a) F *1s* and (b) P *2p*.

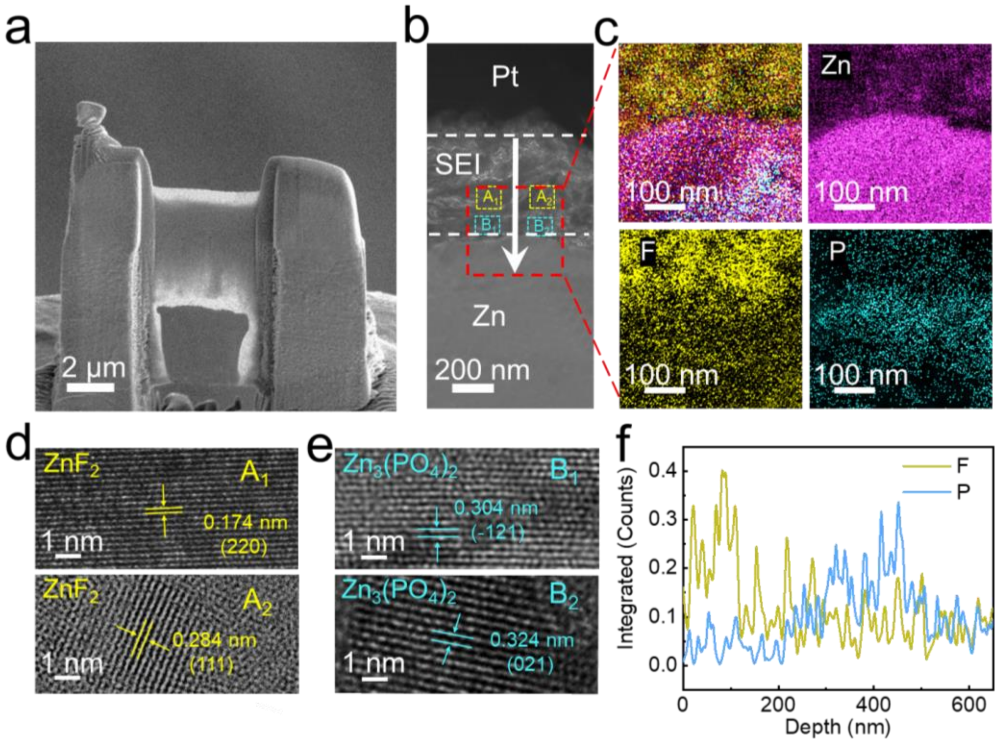

Supplementary Figure 24. Composition analysis of SEI layer formed in TMP–40 electrolyte. (a) SEM image of Zn electrode cycled in the TMP–40 electrolyte after focused ion beam (FIB) cutting. (b) TEM image and (c) EDS mappings of the selected region indicated by a red rectangular box. The Pt deposited on the electrode surface is used to protect the SEI from ion beam damage during sample preparation. (d, e) HRTEM images of the selected regions (A_1, A_2 and B_1, B_2) derived from Supplementary Fig. 24b. (f) EDS line scans of P and F element along the direction of white arrow in Supplementary Fig. 24b.

As displayed in Supplementary Fig. 24a, FIB cutting technique was employed on the Zn electrode cycled in the TMP–40 electrolyte after 20 cycles at a current density of 1 mA cm^{-2} for cross-sectional analysis. Accordingly, the EDS mappings (Supplementary Fig. 24c) in the selected region of Supplementary Fig. 24b reveal that the SEI layer mainly consists of Zn, F, and P elements. Notably, the content of F element predominately distributes the upper region of the as-formed SEI, while the P element mainly concentrates on the top surface of Zn electrode. The HRTEM images of the selected regions of A_1, A_2 and B_1, B_2 derived from Supplementary Fig. 24b confirms the lattice fringes of ZnF_2 and $Zn_3(PO_4)_2$ (Supplementary Fig. 24d, e), further confirming that ZnF_2 and $Zn_3(PO_4)_2$ dominates the top and bottom of the SEI, respectively. Moreover, EDS lines scan along the direction of the arrow in Supplementary Fig. 24b show the F content gradually decreases, while the P content

gradually increases as the depth of SEI increases (Supplementary Fig. 24f), according well with the EDS mappings.

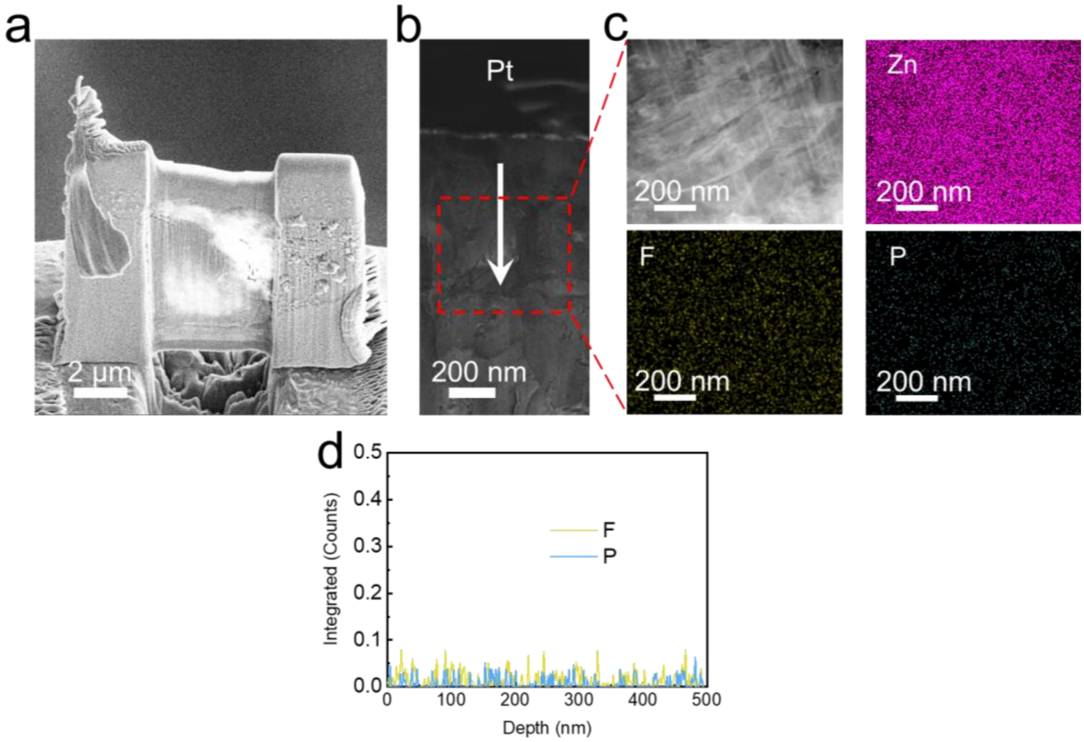

Supplementary Figure 25. Composition analysis of passivation film formed in TMP–0 electrolyte. (a) SEM image of Zn electrode cycled in the TMP–0 electrolyte after FIB cutting for TEM analysis. (b) TEM image and (c) EDS mappings of the selected region indicated by a red rectangular box. The Pt deposited on the electrode surface is used to protect the SEI from ion beam damage during sample preparation. (d) EDS line scans along the direction of white arrow in Supplementary Fig. 25b.

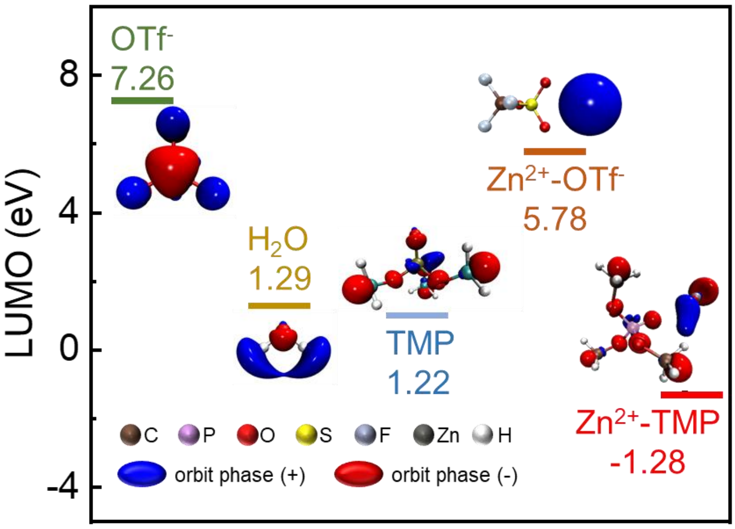

Supplementary Figure 26. LUMO energy levels with corresponding isosurfaces of free OTf^{-}, free TMP, free H_2O, Zn^{2+}–OTf^{-} and Zn^{2+}–TMP coordination.

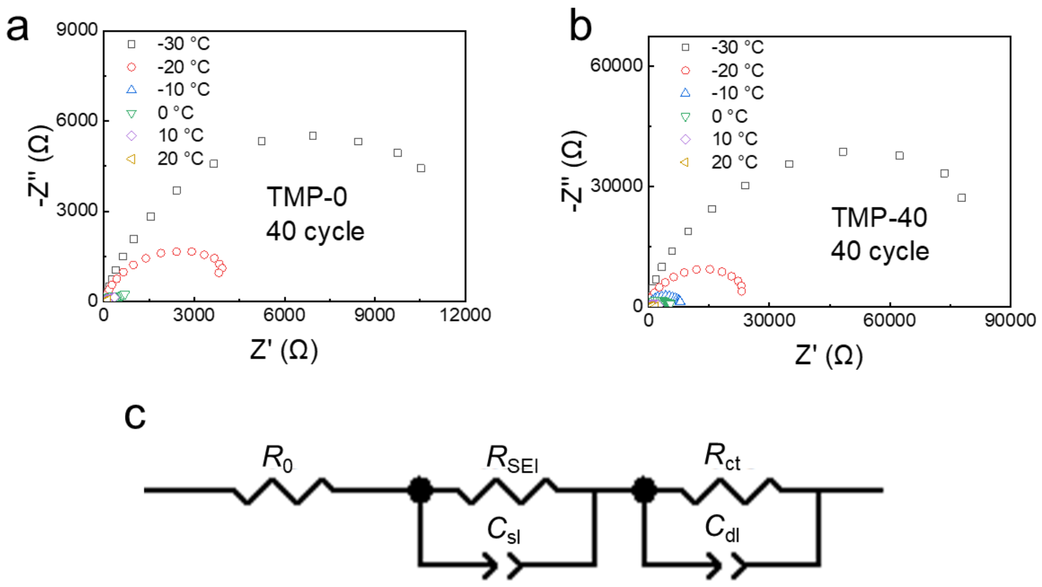

The fitting results corresponding to R_{ct} and R_{SEI} are as follows:

Temperature (K)		243	253	263	273	283	293
TMP–0	R_{ct} (Ω)	13948	4491	1639	385	125	39
	R_{SEI} (Ω)	6115	2820	995	279	100	28
TMP–40	R_{ct} (Ω)	55837	22355	10203	5010	1621	441
	R_{SEI} (Ω)	31926	11203	5456	2356	1047	298

Supplementary Figure 27. Comparison of kinetic performance of SEI after cycling with different electrolytes. Temperature-dependent electrochemical impedance spectra of Zn||Zn symmetric cells after 40 cycles with (a) TMP–0 and (b)TMP–40 electrolyte. (c) The equivalent circuit model. R_0 is bulk resistance of the cell, which reflects electric conductivity of the electrolyte, separator and electrodes; R_{SEI} and C_{sl} are resistance and capacitance of the surface layer, i.e. the SEI film formed on the surface of the electrodes; R_{ct} and C_{dl} are the Faradic charge-transfer resistance and its relative double-layer capacitance, respectively.

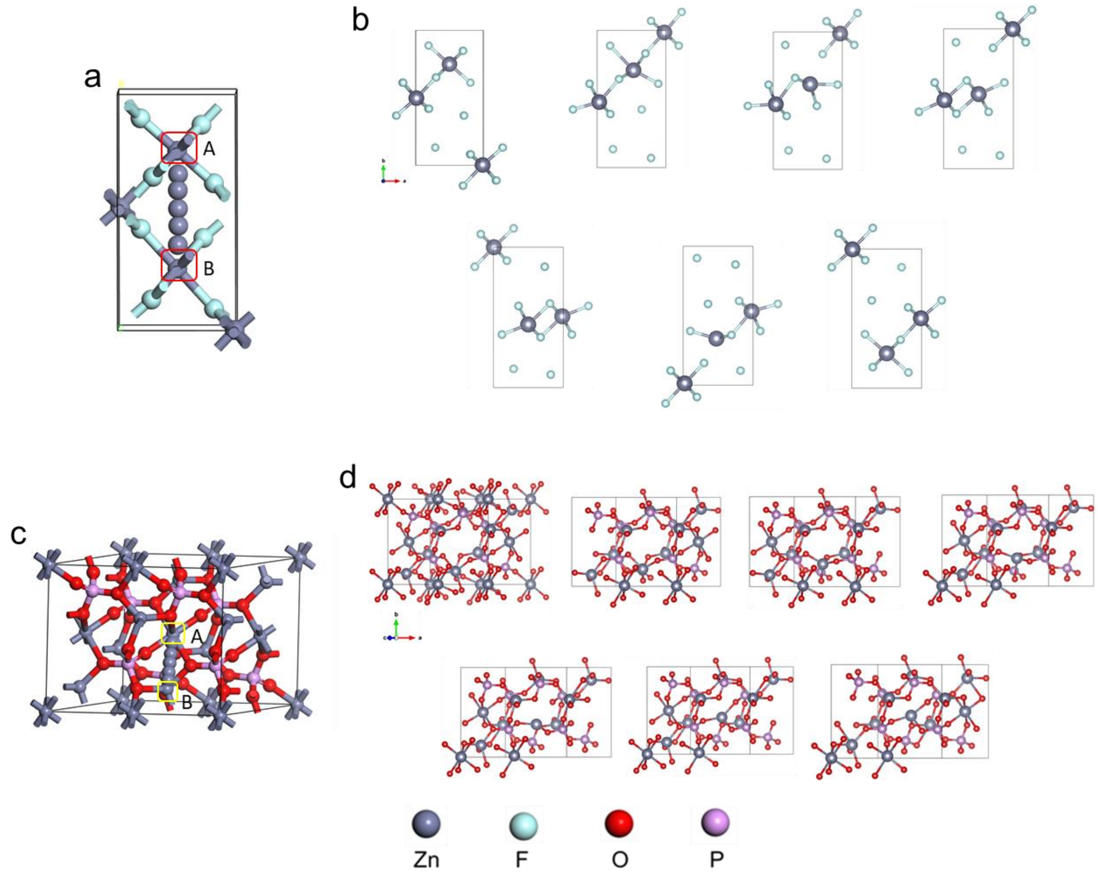

Supplementary Figure 28. Comparison of migration pathways of Zn^{2+} in different SEI components. (a) Simulation of Zn^{2+} migration path in ZnF_2 and (b) corresponding migration models from A to B. (c) Simulation of Zn^{2+} migration path in $Zn_3(PO_4)_2$ and (d) corresponding migration models from A to B.

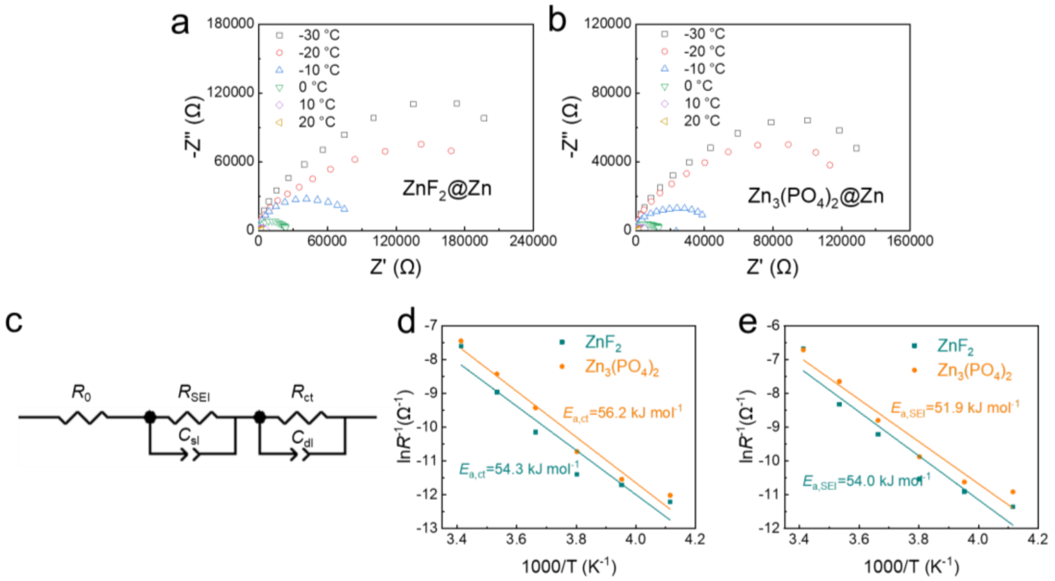

The fitting results corresponding to R_{ct} and R_{SEI} are as follows:

Temperature (K)		243	253	263	273	283	293
ZnF_2	R_{ct} (Ω)	201820	122030	89210	25574	7799	2006
	R_{SEI} (Ω)	85699	54305	37539	9998	4148	807
$Zn_3(PO_4)_2$	R_{ct} (Ω)	165690	103290	45630	12466	4579	1722
	R_{SEI} (Ω)	54755	41025	19520	6634	2100	826

Supplementary Figure 29. Kinetic performance of single ZnF_2 coating Zn metal (ZnF_2@Zn) and single $Zn_3(PO_4)_2$ coating Zn metal ($Zn_3(PO_4)_2$@Zn). Temperature-dependent electrochemical impedance spectra of (a) ZnF_2@Zn||ZnF_2@Zn and (b) $Zn_3(PO_4)_2$@Zn||$Zn_3(PO_4)_2$@Zn. (c) The equivalent circuit model. R_0 is bulk resistance of the cell, which reflects electric conductivity of the electrolyte, separator and electrodes; R_{SEI} and C_{sl} are resistance and capacitance of the surface layer, i.e. the SEI film formed on the surface of the electrodes; R_{ct} and C_{dl} are the Faradic charge-transfer resistance and its relative double-layer capacitance, respectively. Arrhenius fitting of (d) R_{ct} and (e) R_{SEI} derived from the Nyquist plots of the ZnF_2@Zn||ZnF_2@Zn cells and $Zn_3(PO_4)_2$@Zn||$Zn_3(PO_4)_2$@Zn cells.

As shown in Supplementary Fig. 29d, e, the $Zn_3(PO_4)_2$ SEI exhibits higher Zn^{2+}-desolvation energy ($E_{a,ct}$=56.2 kJ mol^{-1}) and lower migration energy barrier of Zn^{2+} ($E_{a,ct}$=51.9 kJ mol^{-1}) compared with ZnF_2 SEI ($E_{a,ct}$=54.3 kJ mol^{-1} and $E_{a,SEI}$=54.0 kJ mol^{-1}). This further confirms the superiority of gradient ZnF_2–$Zn_3(PO_4)_2$ SEI formed by TMP–40 electrolyte.

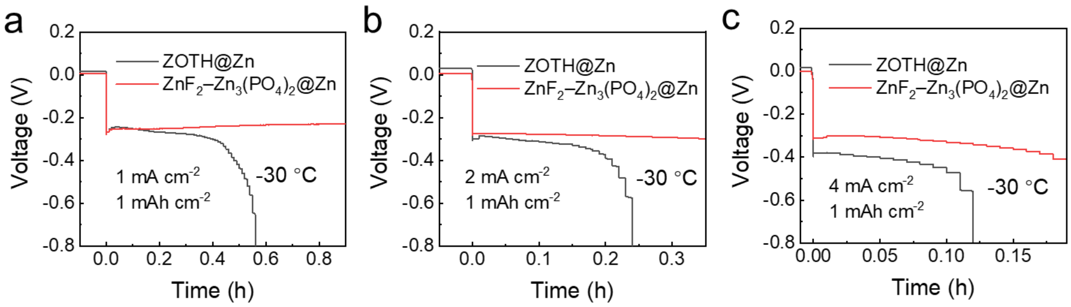

Supplementary Figure 30. Comparison of polarization voltages of Zn||Zn symmetric cells with different SEI components at −30 °C under different current densities. (a) 1 mA cm^{-2}, (b) 2 mA cm^{-2} and (c) 4 mA cm^{-2}.

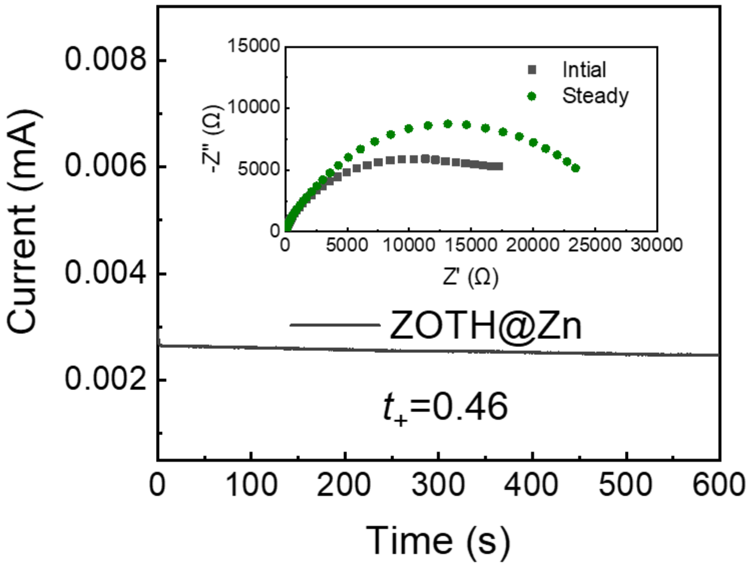

Supplementary Figure 31. Zn^{2+} transference number test for ZOTH@Zn in symmetric cell at −30 °C. Inset: Impedance spectra before and after *i*-*t* testing.

The Zn^{2+} transference number (t_+) was determined as

$$t_+ = \frac{I_S(V\text{-}I_0R_0)}{I_0(V\text{-}I_SR_S)}$$

Where V, I_0 (I_S) and R_0 (R_S) represent applied voltage (10 mV), initial (steady) current response and electrode resistance, respectively. Their values are as follows:

Simple	I_0 (mA)	R_0 (Ω)	I_S (mA)	R_S (Ω)	t_+
ZnF_2–$Zn_3(PO_4)_2$@Zn	8.25×10^{-3}	7600	2.06×10^{-3}	12200	0.87
ZOTH@Zn	2.936×10^{-3}	14800	2.476×10^{-3}	28500	0.46

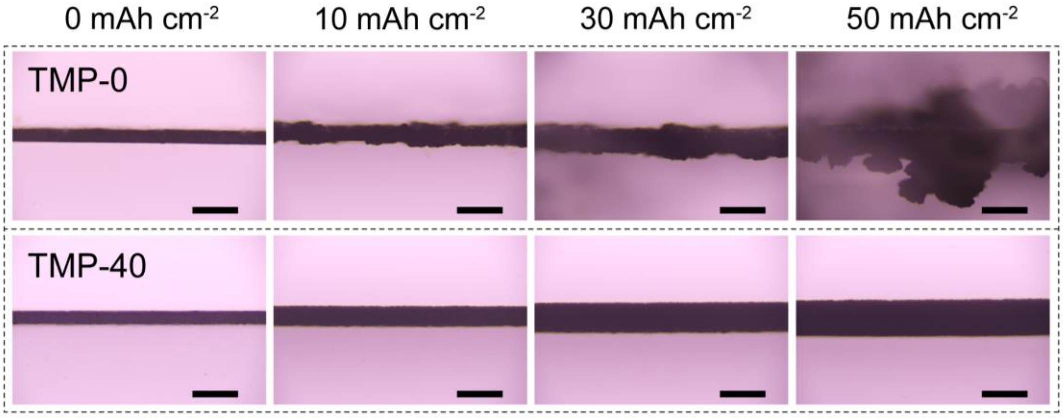

Supplementary Figure 32. In-situ optical observation of Zn deposition process in the electrolyte of TMP–0 (top) and TMP–40 (bottom). The scale bar is 200 μm.

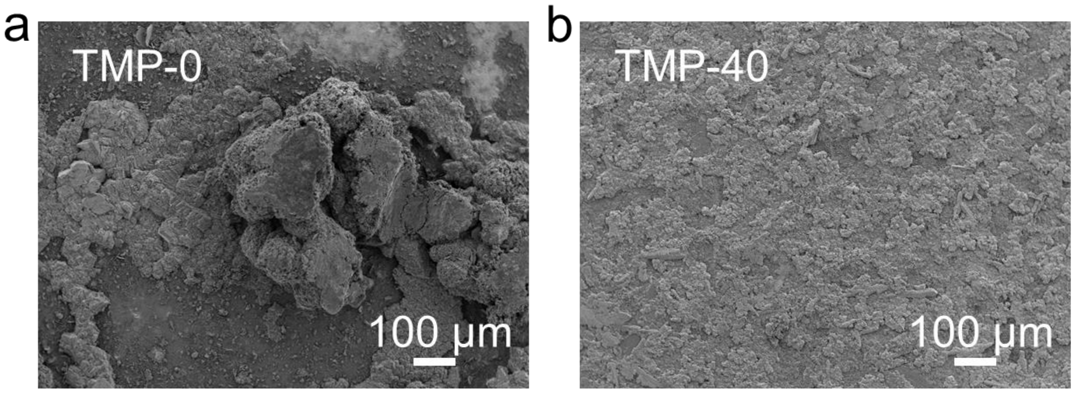

Supplementary Figure 33. Surface morphology after 50 mAh cm^{-2} of deposited Zn in different electrolytes. (a) TMP–0 and (b) TMP–40.

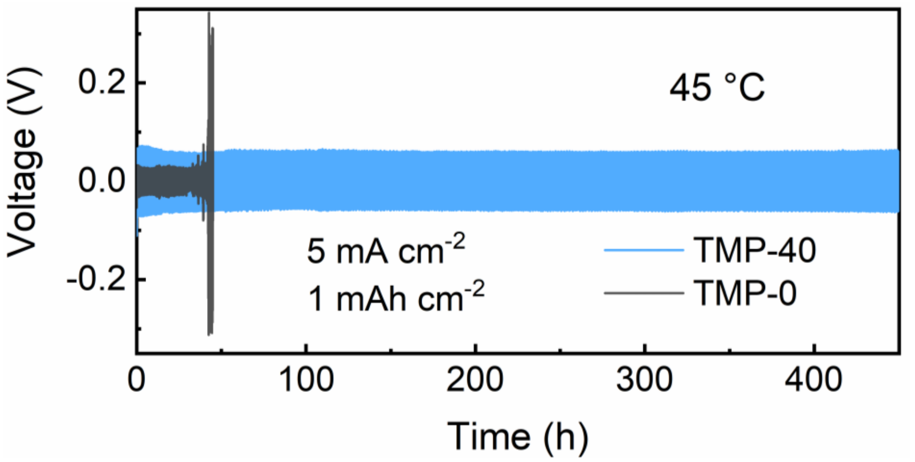

Supplementary Figure 34. Galvanostatic cycling stability of symmetric Zn cells with TMP–0 and TMP–40 electrolytes at 45 °C.

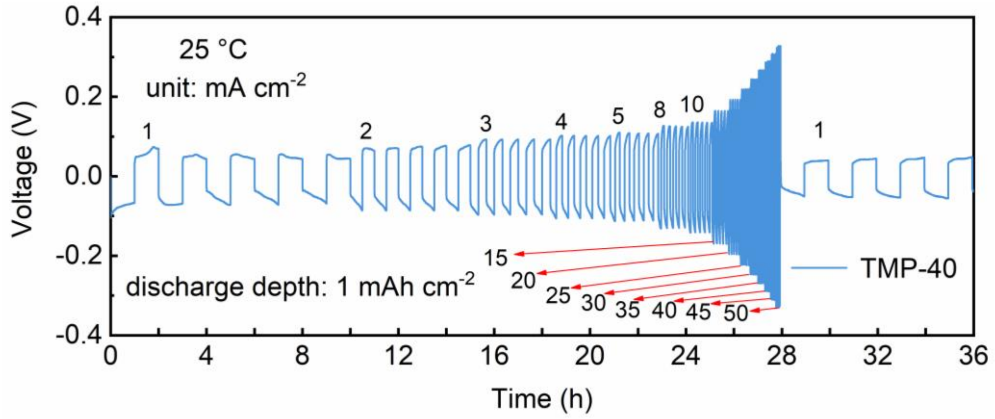

Supplementary Figure 35. Rate performance test of Zn||Zn symmetric cells with TMP–40 electrolyte at 25 °C. The current density varies from 1, 2, 3, 4, 5, 8, 10, 15, 20, 25, 30, 35, 40, 45 to 50 mA cm^{-2} every 5 cycles with a discharge depth fixed at 1 mAh cm^{-2}.

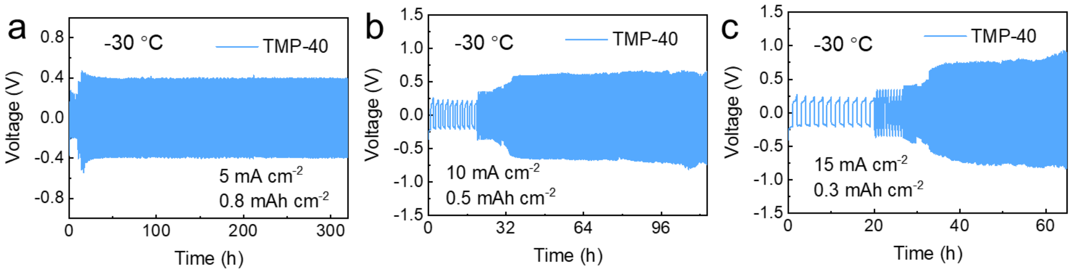

Supplementary Figure 36. Cycling stability of symmetric Zn cells with TMP–40 electrolyte at high current densities under −30 °C. (a) 5 mA cm^{-2} (Pre-activate by 5 cycles at 1 and 3 mA cm^{-2}, respectively), (b) 10 mA cm^{-2} (Pre-activate by 10 cycles at 1, 3, 5 and 8 mA cm^{-2}, respectively) and (c) 15 mA cm^{-2} (Pre-activate by 10 cycles at 1, 3, 5, 8, 10 and 12 mA cm^{-2}, respectively).

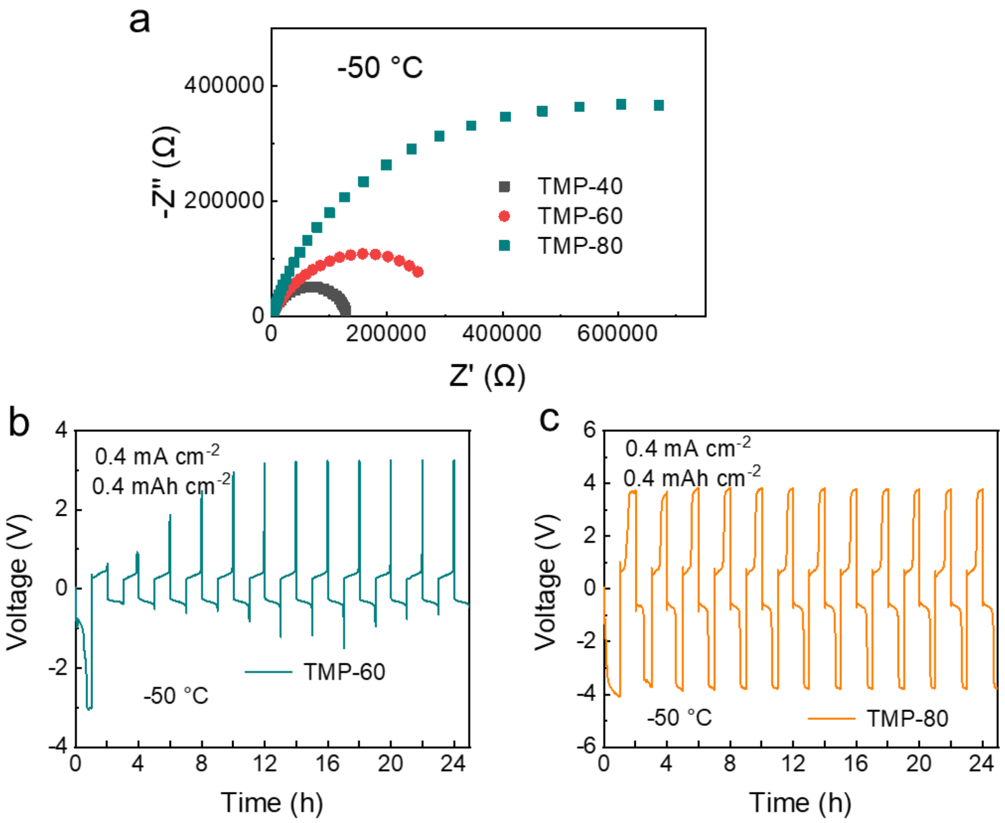

Supplementary Figure 37. Electrochemical performance comparison of different electrolytes at −50 °C. (a) Comparison of electrochemical impedance spectra of different electrolytes at −50 °C. Cycling stability of symmetric Zn cells with (b) TMP–60 and (c) TMP–80 electrolyte at 0.4 mA cm^{-2} 0.4 mAh cm^{-2} under a low temperature of −50 °C.

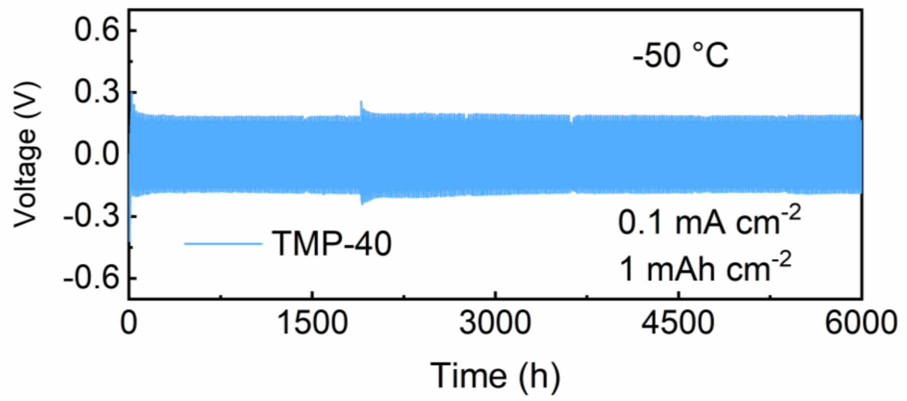

Supplementary Figure 38. Galvanostatic cycling stability of symmetric Zn cells with TMP–40 electrolyte at 0.1 mA cm^{-2} and 1 mAh cm^{-2} at −50 °C.

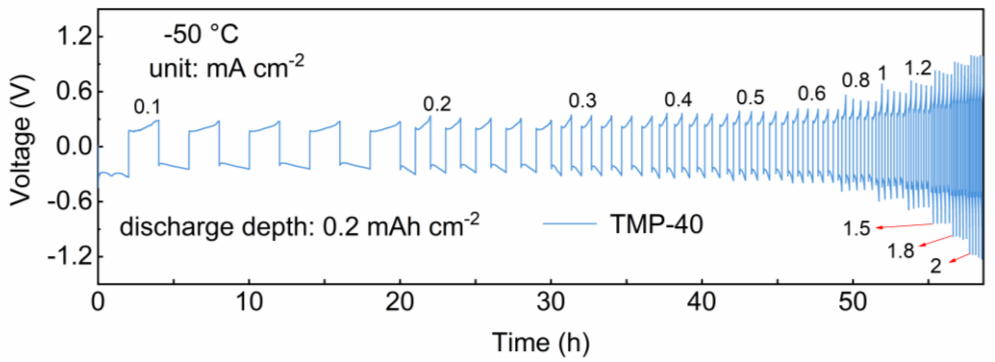

Supplementary Figure 39. Rate performance test of Zn||Zn symmetric cells with TMP–40 electrolyte at −50 °C. The current density varies from 0.1, 0.2, 0.3, 0.4, 0.5, 0.6, 0.8, 1, 1.2, 1.5, 1.8 to 2 mA cm^{-2} every 5 cycles with a discharge depth fixed at 0.2 mAh cm^{-2}.

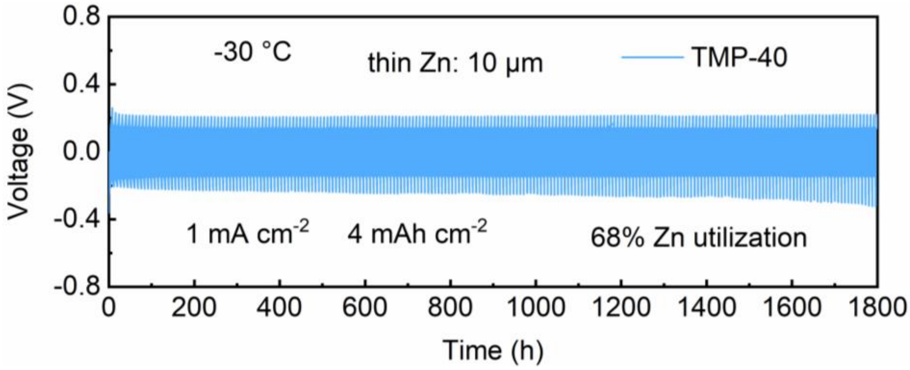

Supplementary Figure 40. Galvanostatic cycling stability of symmetric Zn cells in TMP–40 with a Zn utilization of 68% at −30 °C.

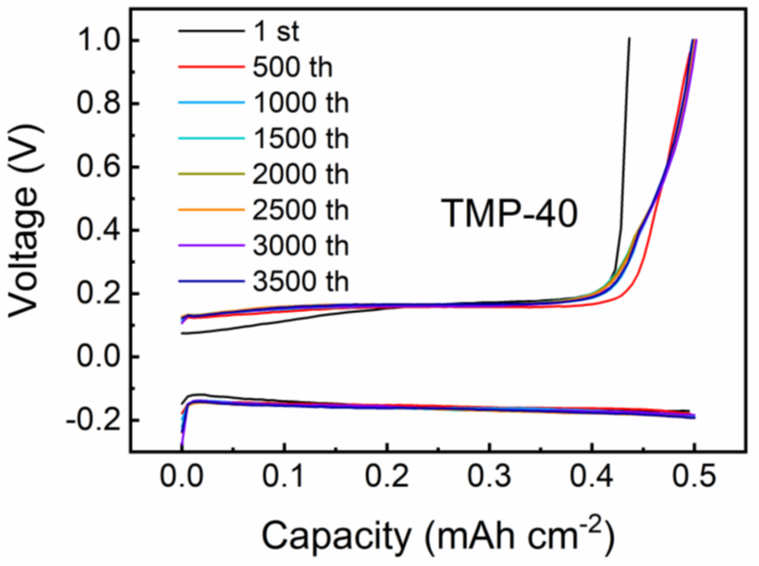

Supplementary Figure 41. Voltage profiles of Zn deposition on Ti substrate at a current density of 1 mA cm^{-2} and capacity of 0.5 mAh cm^{-2} after different cycles at −30 °C.

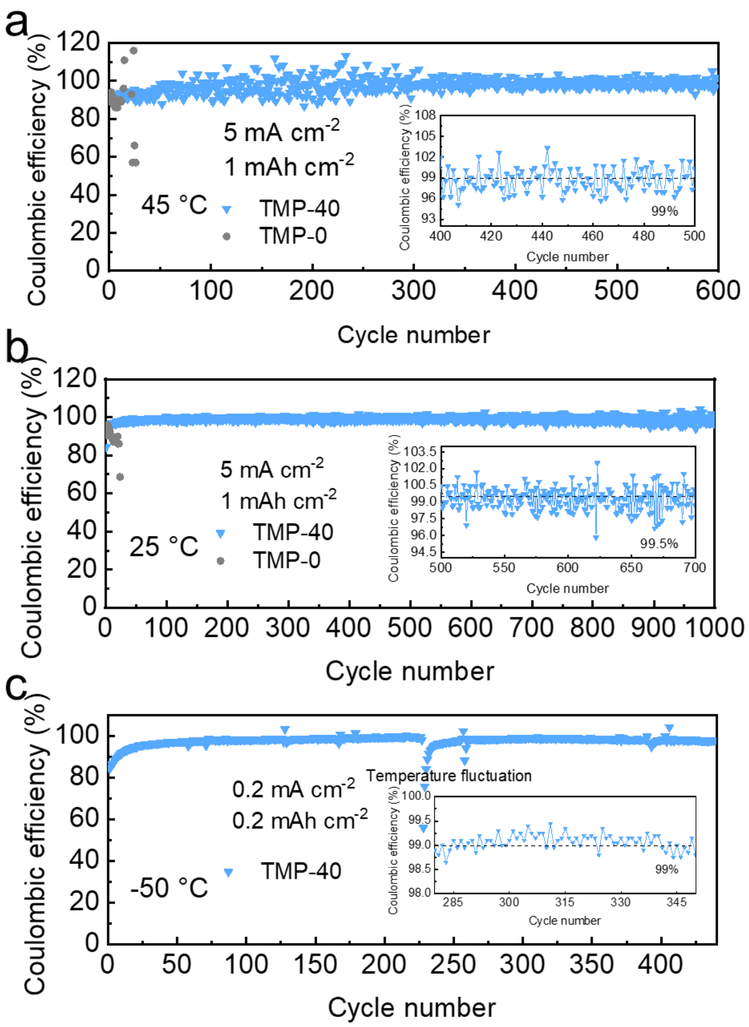

Supplementary Figure 42. Long-term Zn plating/stripping Coulombic efficiency with TMP–40 and TMP–0 electrolytes at different temperatures. (a) 45 °C, (b) 25 °C and (c) −50 °C.

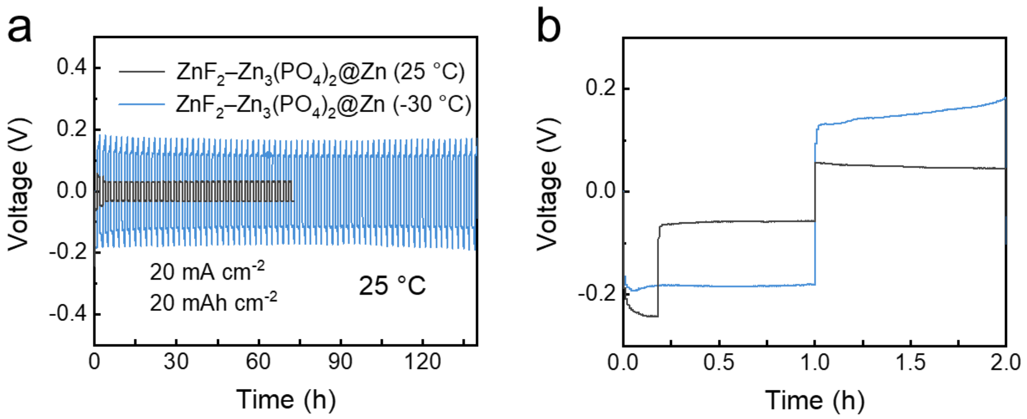

Supplementary Figure 43. Comparison of Zn plating/stripping stability for SEI formation at different temperatures. (a) Galvanostatic cycling stability of ZnF_2–$Zn_3(PO_4)_2$@Zn||ZnF_2–$Zn_3(PO_4)_2$@Zn (25 °C) and ZnF_2–$Zn_3(PO_4)_2$@Zn||ZnF_2–$Zn_3(PO_4)_2$@Zn (−30 °C) symmetric cells with a current density of 20 mA cm^{-2} and discharge depth of 20 mAh cm^{-2} at 25 °C. (b) The enlarged voltage profiles at the first cycle.

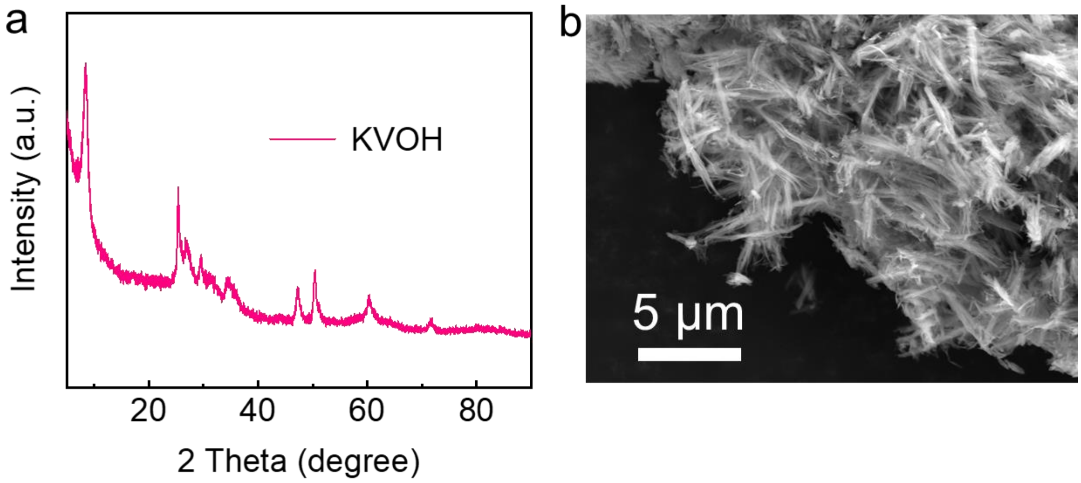

Supplementary Figure 44. Structure and morphology characterization of KVOH. (a) XRD pattern and (b) SEM image.

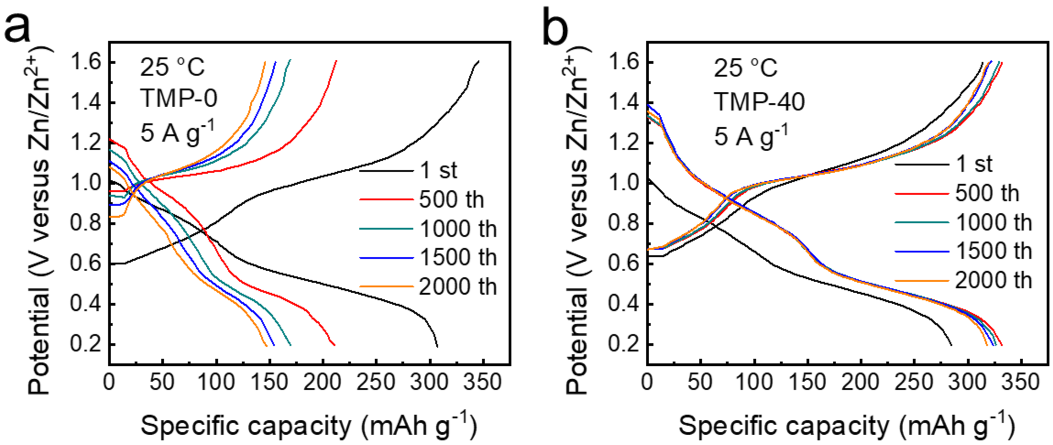

Supplementary Figure 45. Comparison of charge–discharge curves with different cycles using different electrolytes. (a) TMP–0 and (b) TMP–40.

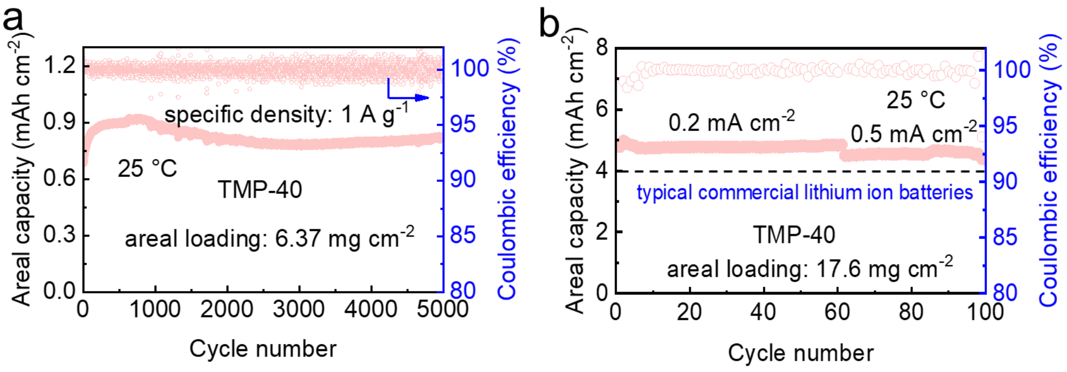

Supplementary Figure 46. The cycling performance of Zn–KVOH full cells in the TMP–40 electrolyte with different cathode areal loadings. (a) 6.37 mg cm^{-2} (The cell was pre-activated by performing 3 cycles with small specific currents of 0.1, 0.2, and 0.5 A g^{-1}, respectively) and (b) 17.6 mg cm^{-2}.

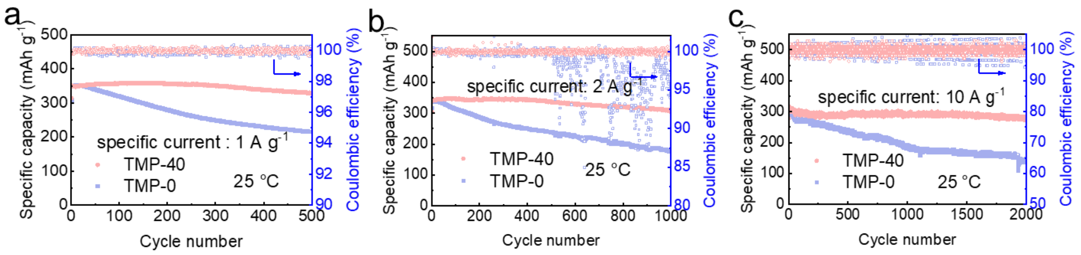

Supplementary Figure 47. The cycling performance of Zn–KVOH full cells with different specific currents at 25 °C. (a) 1A g^{-1}, (b) 2 A g^{-1} and (c) 10 A g^{-1}.

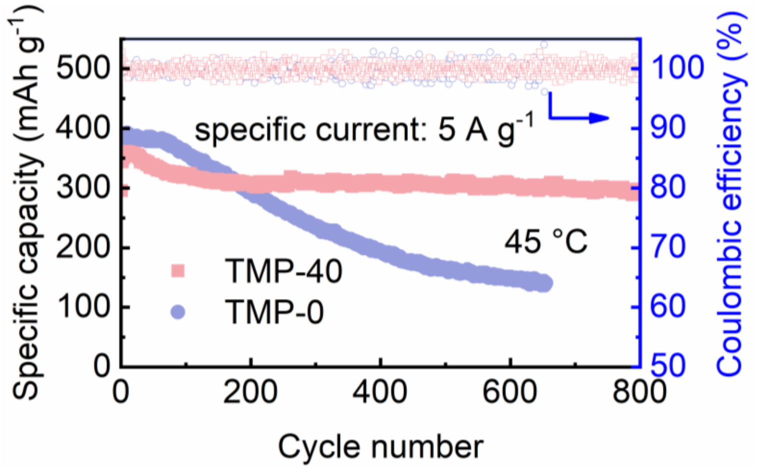

Supplementary Figure 48. The cycling performance of Zn–KVOH full cells with TMP–40 and TMP–0 electrolytes at 45 °C.

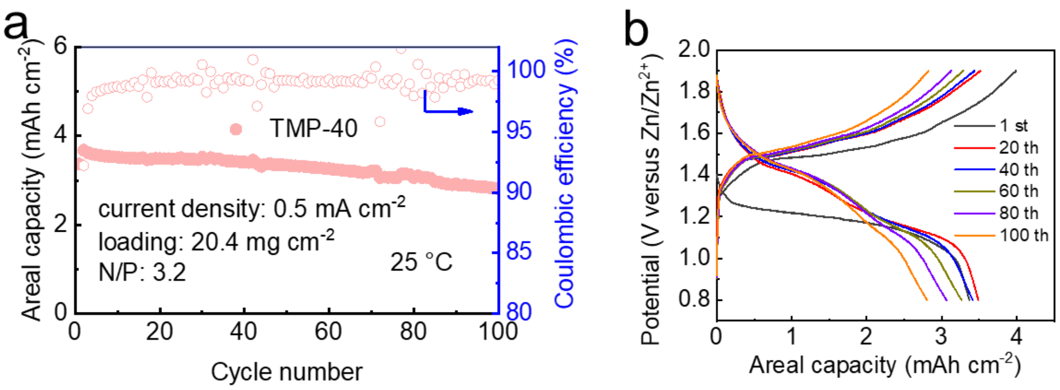

Supplementary Figure 49. Zn–MnO_2 full cell test under practical conditions (high loading: 20.4 mg cm^{-2}, low N/P: 3.2) with TMP–40 electrolyte. (a) Cycling performance, (b) charge–discharge curves with different cycles.

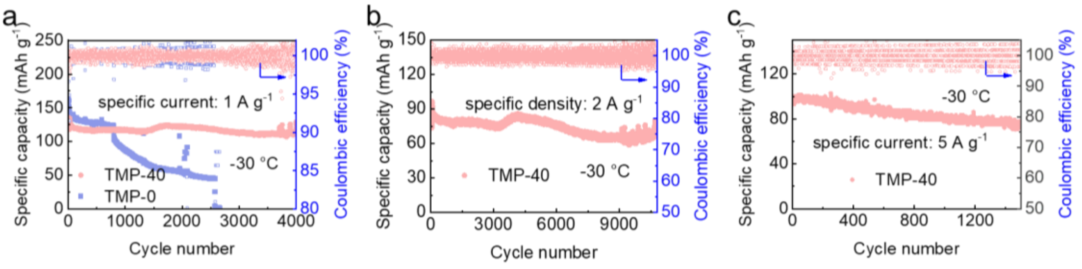

Supplementary Figure 50. The cycling performance of Zn–KVOH full cells with different specific currents at −30 °C. (a) 1A g^{-1}, (b) 2 A g^{-1} and (c) 5 A g^{-1}.

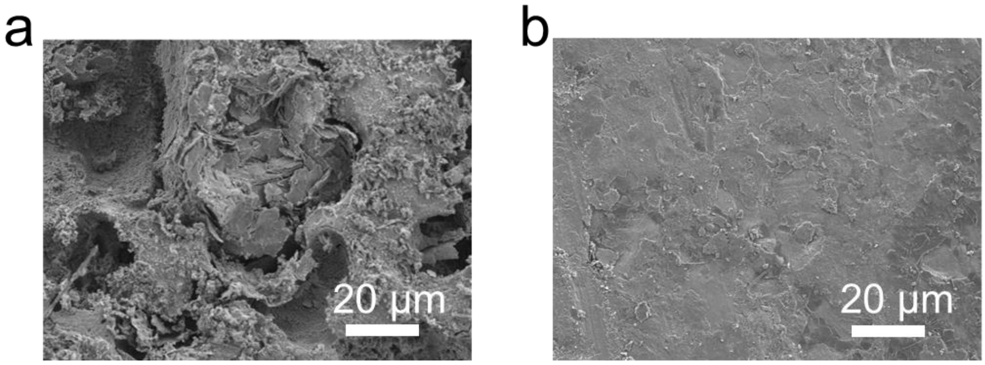

Supplementary Figure 51. Surface morphology of Zn anode dissembled from Zn–KVOH full cell after 1000 cycles in different electrolytes. (a) TMP–0 and (b) TMP–40.

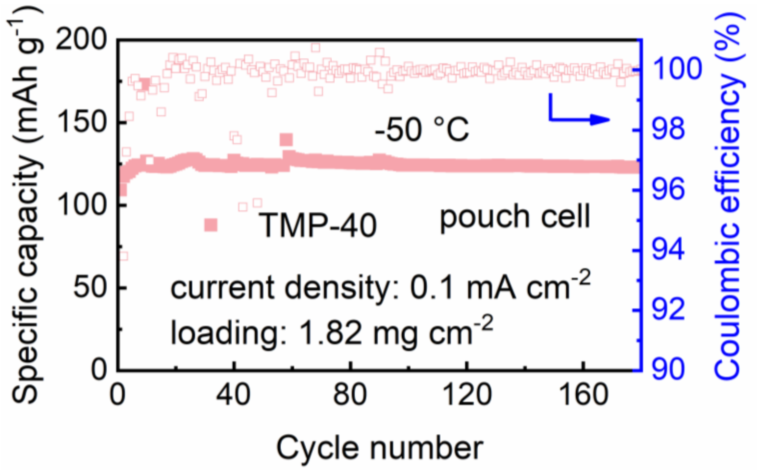

Supplementary Figure 52. Cycling performance of Zn–KVOH pouch cell in TMP–40 electrolyte at −50 °C.

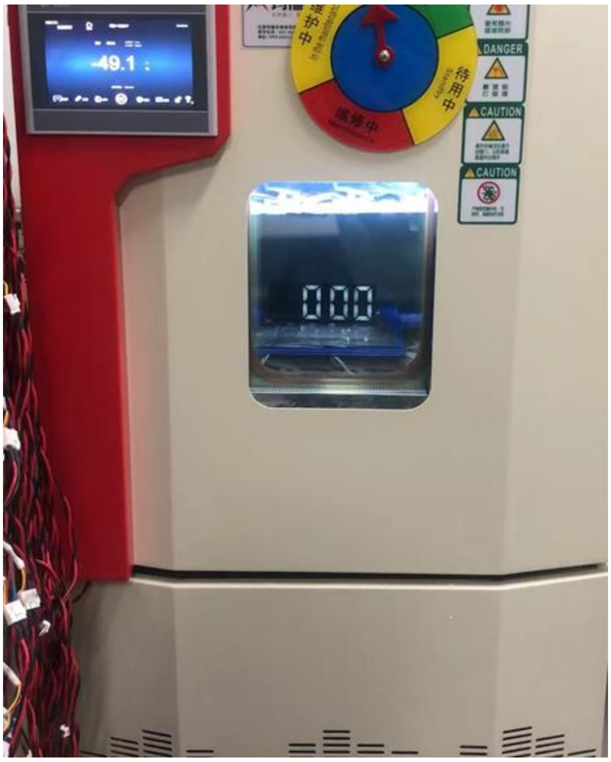

Supplementary Figure 53. Photograph of the pouch cells in the cryostat at −50 °C.

Supplementary Table 1. The ionic conductivities of different electrolytes at various temperatures.

Temperature (°C)	Ionic conductivity (mS cm^{-1})							
	TMP–0	TMP–5	TMP–10	TMP–20	TMP–40	TMP–60	TMP–80	TMP–100
60	200	172.41	160.41	138.50	83.75	46.81	15.60	5.72
40	132.45	125.73	121.06	105.04	44.23	30.01	8.41	4.22
20	88.26	87.71	86.28	73.63	43.29	18.03	4.16	1.83
10	68.96	63.73	62.26	53.70	32.52	12.59	2.68	1.09
0	51.54	47.84	44.32	37.50	23.00	8.67	1.54	0.59
−10	37.59	31.99	29.56	24.73	14.97	5.24	0.80	0.29
−20	23.11	20.16	17.75	15.41	9.22	3.05	0.41	0.12
−30	12.41	10.39	9.36	8.53	4.81	1.51	0.16	0.004
−40	8.33E-5	2.80	2.62	3.52	2.24	0.66	0.052	0.001
−50	1.11E-5	0.67	0.62	0.65	0.85	0.24	0.012	4.07E-4
−60	-	0.11	0.10	0.12	0.18	0.096	0.002	1.35E-4

Supplementary Table 2. Numbers of ions and solvent molecules in the investigated simulation systems.

System	Zn^{2+}	$OTf^{–}$	TMP	Water
TMP–0	70	140	0	1945
TMP–40	70	140	122	1167

Supplementary Table 3. The summary of cumulative capacity of different reports under wide temperature range.

Refs	Temperature (°C)	Cumulative capacity (mAh cm^{-2})
This work	45	1125
	25	1250
	−30	3600
	−50	1400
1	20	150
	−20	80
2	25	62.5
	0	50
	−30	375
3	−20	2500
	−40	500
	−50	50
4	25	750
	−30	500
5	50	800
	25	1000
	0	1000
	−15	150
6	60	400
	20	500
	−20	100

Supplementary Table 4. Summary of the cycling performance of the full cells with TMP–0 or TMP–40 electrolyte at different temperatures.

Temperature (°C)	Electrolyte	Specific current (A g^{-1})	Cycle number	Capacity retention (%)
45	TMP–40	5	800	85.7
	TMP–0	5	655	36.5
25	TMP–40	1	500	93.8
		2	1000	89.6
		5	2300	98.3
		10	2000	90.0
	TMP–0	1	500	61.8
		2	1000	52.0
		5	2300	41.1
		10	2000	46.4
−30	TMP–40	1	4000	88.0
		2	10800	73.3
		5	1500	73.6
	TMP–0	1	2664	1.2
−50	TMP–40	0.5	12000	86.1

Supplementary Table 5. Summary of the cycle performance of the full cells with various published works on electrolyte modification for aqueous Zn batteries.

Refs	Temperature (°C)	Discharge capacity (mAh g^{-1})	Specific current (A g^{-1})	Cycle number	Capacity decay rate per cycle (%)
This work	45	343.4	5	800	0.018
	25	329.1	5	2300	0.00074
	−30	95.5	2	10800	0.0025
	−50	59	0.5	12000	0.0012
1	−20	~100	0.2	250	0.0080
3	−40	69	1	1000	~0.0069
4	25	80	5	2000	~0.010
	−30	70	5	2500	~0.0060
5	50	~100	--	200	0.34
	25	103.9	--	600	0.051
	0	77.8	--	1000	0.048
	−15	~80	0.1	450	~0
6	25	290	2	1500	0.0040
	−30	131	0.1	300	0.066
7	25	~150	1	800	0.050
	−15	~110	0.5	300	0.052
8	−50	105.7	~0.05	200	0.025
9	20	220	~0.3	500	0.16
	−30	175	~0.3	1000	0.020
10	25	~170	2.6	500	0.0094
11	25	~140	5	2000	0.0072
	−10	~110	5	2000	0.0054
12	25	230	1	500	0.032
13	60	200	2	100	~0.54
	20	82	2	1000	~0
	−20	~60	0.2	1000	~0
14	25	265.6	1	1000	0.020
15	25	160	6	1000	~0
	−20	65	6	1000	~0
	−50	40	6	1000	~0
16	30	229	5	1000	0.0030
	−40	168	0.2	400	~0

Supplementary References

1. Chang, N. N. et al. An aqueous hybrid electrolyte for low-temperature zinc-based energy storage devices. *Energy Environ. Sci.* **13**, 3527–3535 (2020).

2. Sun, T. J. et al. An ultralow-temperature aqueous zinc-ion battery. *J. Mater. Chem. A* **9**, 7042–7047 (2021).

3. Wang, J. W. et al. Low-temperature and high-rate Zn metal batteries enabled by mitigating Zn^{2+} concentration polarization. *Chem. Eng. J.* **433**, 134589 (2022).

4. Huang, S. W. et al. Antifreezing hydrogel electrolyte with ternary hydrogen bonding for high-performance zinc-ion batteries. *Adv. Mater.* **34**, 2110140 (2022).

5. Hou, Z., Lu, Z. H., Chen, Q. W. & Zhang, B. Realizing wide-temperature Zn metal anodes through concurrent interface stability regulation and solvation structure modulation. *Energy Storage Mater.* **42**, 517–525 (2021).

6. Zhou, J. et al. Highly Reversible and Stable Zn Metal Anode under Wide Temperature Conditions Enabled by Modulating Electrolyte Chemistry. *Chem. Eng. J.* **442**, 136218 (2022).

7. Han, D. L. et al. A non-flammable hydrous organic electrolyte for sustainable zinc batteries. *Nat. Sustain.* **5**, 205–213 (2022).

8. Cao, L. S. et al. Highly reversible aqueous zinc batteries enabled by zincophilic–zincophobic interfacial layers and interrupted hydrogen-bond electrolytes. *Angew. Chem. Int. Ed.* **60**, 18845–18851 (2021).

9. Gao, S. Y. et al. High energy and stable subfreezing aqueous Zn-MnO_2 batteries with selective and pseudocapacitive Zn-ion insertion in MnO_2. *Adv. Mater.* **34**, 2201510 (2022).

10. Cao, L. S. et al. Solvation structure design for aqueous Zn metal batteries. *J. Am. Chem. Soc.* **142**, 21404–21409 (2020).

11. Hao, J. N. et al. Boosting zinc electrode reversibility in aqueous electrolytes by using low-cost antisolvents. *Angew. Chem. Int. Ed.* **60**, 7366–7375 (2021).

12. Han, D. L. et al. A self-regulated interface toward highly reversible aqueous zinc batteries. *Adv. Energy Mater.* **12**, 2102982 (2022).

13. Du, H. H. et al. Improving zinc anode reversibility by hydrogen bond in hybrid aqueous electrolyte. *Chem. Eng. J.* **427**, 131705 (2022).

14. Sun, P. et al. Simultaneous Regulation on Solvation Shell and Electrode Interface for Dendrite-free Zn ion batteries achieved by a low-cost glucose additive. *Angew. Chem. Int. Ed.* **60**, 18247–18255 (2021).
15. Yang, G. S. et al. An aqueous zinc-ion battery working at −50 °C enabled by low-concentration perchlorate-based chaotropic salt electrolyte. *Ecomat* **4**, e12165 (2022).
16. Liu, D. S. et al. Regulating the electrolyte solvation structure enables ultralong lifespan Vanadium-based cathodes with excellent low-temperature Performance. *Adv. Funct. Mater.* **32**, 2111714 (2022).
